# The G protein-coupled receptors in the pufferfish *Takifugu rubripes*

**DOI:** 10.1186/1471-2105-12-S1-S3

**Published:** 2011-02-15

**Authors:** Anita Sarkar, Sonu Kumar, Durai Sundar

**Affiliations:** 1 Department of Biochemical Engineering and Biotechnology, Indian Institute of Technology (IIT) Delhi, New Delhi, India

## Abstract

**Background:**

Guanine protein-coupled receptors (GPCRs) constitute a eukaryotic transmembrane protein family and function as “molecular switches” in the second messenger cascades and are found in all organisms between yeast and humans. They form the single, biggest drug-target family due to their versatility of action and their role in several physiological functions, being active players in detecting the presence of light, a variety of smells and tastes, amino acids, nucleotides, lipids, chemicals etc. in the environment of the cell. Comparative genomic studies on model organisms provide information on target receptors in humans and their function. The Japanese teleost Fugu has been identified as one of the smallest vertebrate genomes and a compact model to study the human genome, owing to the great similarity in its gene repertoire with that of human and other vertebrates. Thus the characterization of the GPCRs of Fugu would provide insights to the evolution of the vertebrate genome.

**Results:**

We classified the GPCRs in the Fugu genome and our analysis of its 316 membrane-bound receptors, available on the public databases as well as from literature, detected 298 GPCRs that were grouped into five main families according to the GRAFS classification system (namely, Glutamate, Rhodopsin, Adhesion, Frizzled and Secretin). We also identified 18 other GPCRs that could not be grouped under the GRAFS family and hence were classified as ‘Other 7TM’ receptors. On comparison of the GPCR information from the Fugu genome with those in the human and chicken genomes, we detected 96.83% (306/316) and 96.51% (305/316) orthology in GPCRs among the Fugu-human genomes and Fugu-chicken genomes, respectively.

**Conclusions:**

This study reveals the position of pisces in vertebrate evolution from the GPCR perspective. Fugu can act as a reference model for the human genome for other protein families as well, going by the high orthology observed for GPCRs between Fugu and human. The evolutionary comparison of GPCR sequences between key vertebrate classes of mammals, birds and fish will help in identifying key functional residues and motifs so as to fill in the blanks in the evolution of GPCRs in vertebrates.

## Background

G protein-coupled receptors (GPCRs) constitute one of the largest and most ancient super-families of integral trans-membrane receptor proteins that act as cellular receptors and play a central role in the signal transduction in eukaryotes [1-4]. They are perhaps the most versatile of all the known proteins in the human genome [5] and hence are the focus of intense pharmaceutical and academic research [6] as they form one of the prime transduction pathways through which acceptance of a signal by a cell can principally occur, the other being tyrosine kinase receptors [7]. Abnormalities in cell signaling by GPCRs are the root cause of wide-spread disorders like asthma, peptic ulcers, hypertension, type II diabetes etc. [8]. GPCRs have triumphed throughout the course of evolution and can probably be traced back to a time before the divergence of plants and animals [5] and possibly even exist in protozoa, though not characterized, yet [9]. Their importance can be gauged from their presence as well as abundance in almost all eukaryotic genomes [10].

The availability of complete sets of putative members of a family from diverse species provides the basis for cross-genome comparative studies. The Japanese pufferfish, *Takifugu rubripes*, having a genome that is one-eighth the size of that found in human, is among the smallest vertebrate genomes and thus an apt reference for annotating the human genome. Teleosts diverged about 500 million years ago from other phyla and are ancestors of tetrapods. Comparing the genomes of Fugu and the green spotted pufferfish (*Tetraodon nigroviridis*), it is observed that about 75% of the duplication events appear to have happened before the *Takifugu* and *Tetraodon* lineages separated [11]. Both these teleost species serve as model genomes but are non-redundant since the two, being separated by approximately 18 to 30 million years, provide an improved annotation on both sides.

We have classified the GPCRs present in the *Takifugu rubripes* genome according to the GRAFS classification system and compared it to GPCRs present in the human, chicken and the *Tetraodon nigroviridis* genomes, which has added to the information of GPCR evolution alongside vertebrate evolution (from data available from the non-redundant public databases) and detected very high orthology between Fugu-human and Fugu-chicken. Additionally we also compared the GPCRs between the Japanese and the green spotted pufferfish for perspective and found that the two pufferfishes had comparable receptors in this superfamily of proteins.

## Results and discussion

**Figure 1:**
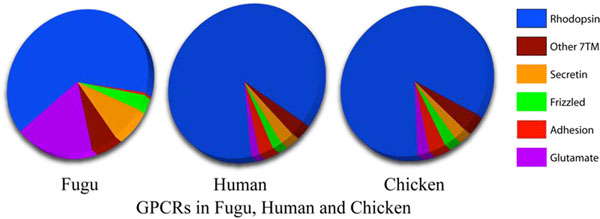
**GPCR distribution in Fugu, Chicken and Human:** The figure illustrates a comparative account of the GPCRs present in the representatives of 3 vertebrate classes.

Our primary objective was to classify the GPCRs of the Fugu (Japanese or tiger pufferfish) genome according to the GRAFS classification system [12], that were available with the non-redundant public databases of NCBI [13] and UniProtKB [14] (where A-F classification system is followed [15]) and from previously published literature [2], as the majority of GPCRs in all eukaryotes, starting from the nematodes in the evolutionary ladder, can be categorized in lieu of the GRAFS system (Figure 1). We also compared the GPCRs between the two puffer fishes to understand the pattern of evolution among teleosts that has been characterized according to GRAFS [11].

Fugu comprises fewer GPCRs than either human and chicken (Table 1). Also our dataset has 150 GPCRs less than that reported in *Tetraodon nigroviridis*[11]. Except for 10 Fugu receptors, all others find matches in the human GPCRs, while all but 11 find counterparts in the chicken GPCR sub-set. All Fugu GPCRs found orthologs in *Tetraodon nigroviridis* though, unlike Fugu, most *Tetraodon* GPCR sequences in the public databases still did not have a clearly defined function. We observed that there are some unique receptors in Fugu which are not present in humans like the pheromone receptors, while some are conspicuous with their absence in Fugu, such as the melanocortin3 (MC3) receptors which are found in human. 18 Fugu receptors did not confirm to the requirements of the GRAFS classification and hence were grouped under the Other 7TMs. For better understanding and clarity in viewing the phylogenetic tree, we grouped the families as Rhodopsins and Non-Rhodopsins (Table 2). A very high orthology was detected between the receptors of chicken and human when compared to those in Fugu (Table 3), keeping with the attribute of Fugu as a reference model for the human genome. Also all Fugu GPCRs found counterparts in *Tetraodon nigroviridis* (Table 2), though the orphans could not be compared between the sub-families as this detail was not found for *Tetraodon nigroviridis*.

**Table 1:** GPCRs in Fugu, Human, Chicken and *Tetraodon nigroviridis*

Receptor family	Fugu	Human	Chicken	***Tetraodon nigroviridis***	
Glutamate	58	15	15	36	
Rhodopsin α β γ δ	110 20 14 60	89 35 59 518	92 45 46 290	137 88 42 75	+ 26 orphans
Adhesion	2	24	22	29	
Frizzled/Tas2	10	24	14	12	
Secretin	24	15	14	21	
Other 7TM	18	23	19	-	

**Table 2:** Rhodopsin and Non-Rhodopsin receptors in Fugu, Human, Chicken and *Tetraodon nigroviridis*

Receptor family	Fugu	Human	Chicken	***Tetraodon nigroviridis***
Non-Rhodopsin	112	101	62	98
Rhodopsin				
Olfactory	56	460	229	22
Non-olfactory	148	241	61	346

**Table 3:** Percentage orthology of Fugu GPCRs with Human and Chicken

Organism	Number of GPCRs orthologous to Fugu	Percentage orthology*
Human	306	96.83
Chicken	305	96.51
Fugu	316	N.A.

* The data includes all those sequences that are homologous to the GPCRs in the Human and Chicken GPCR sub-sets

## Non-Rhodopsins

### Glutamate (58 receptors)

**Figure 2:**
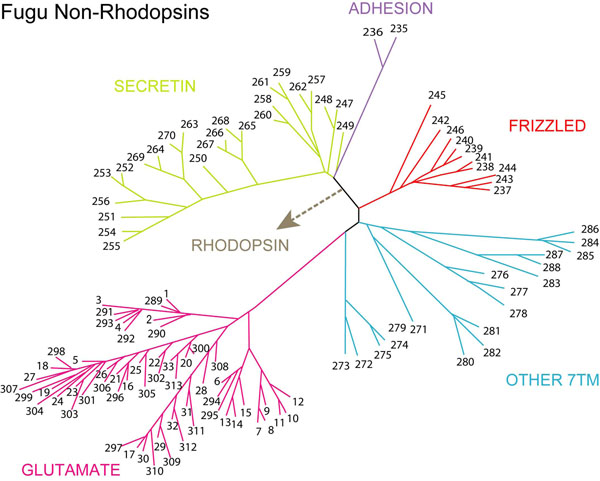
**Phylogenetic tree of Fugu Non-Rhodopsins.** The figure illustrates the members of the Glutamate, Adhesion, Frizzled, Secretin families in Fugu calculated using the neighbor joining (NJ) method based on 1000 replicas of the dataset. The position of the Non-Rhosopsins was established using the 112 receptors of the Fugu non-rhodopsin families. The receptors which lack sequence similarity with any other GPCR family have been categorized as ‘Other 7TM’ and depicted in the phylogenetic tree. The members have been denoted as numerals as per the numbering in Dataset S3.

Figure 2 describes the repertoire of the Fugu GPCRs grouped together as the Non-Rhodopsins, which includes Glutamate, Adhesion, Frizzled, Secretin and Other 7TM families. In our study, we observe that out of the 58 Glutamate receptors in Fugu, all found orthologs in the *Tetraodon*, human and chicken GPCRs but these did not necessarily have the same function. At the sub-family level, the 9 Fugu metabotropic Glutamate (GRM) and the lone Ca^2+^-sensing (CASR) showed conserved function but this was not true for the 36 V2Rs (pheromone receptors of Fugu) which are absent in human and chicken. The V2Rs found orthologs in the CASR and GRM receptors of chicken and human. The 12 type 1 taste (TAS1) receptors in Fugu matched with various other members of the type 1 receptors in both human and chicken. Interestingly, the corresponding receptors were exactly the same in human and chicken, which occurs probably due to an evolutionary diversification at a stage later than pisces in the course of vertebrate evolution. The presence of TAS1 receptors in both the pufferfishes points to the presence of chemosensory glutamate receptors prior to the evolution of land vertebrates. Like the chicken GPCR subset, Fugu also seems to lack the ortholog for the GABABR (gamma aminobutyric acid-binding receptor) though 4 such representatives were detected in *Tetraodon nigroviridis*. Probably, this is observed due to a different mode of function of GABA receptors in Fugu and chicken as compared to *Tetraodon nigroviridis* and human.

### Adhesion (2 receptors)

The adhesion sub-family is known to have immunological functions as well as plays important roles in the central nervous system [16]. They are unique in comprising of certain motifs, rich in glycosylation sites and proline residues, occurring in the long (200-800 amino acids) N-termini, that are likely-players in cell adhesion [17,18]. This family has been referred to by many other names due to some other unique characteristics, for instance, it has been called EGF-TM7 to emphasise the presence of epidermal growth factors (EGF) domains in its N-termini, LN-TM7 receptors, due to its long variable N-termini, as already mentioned above, and also termed B2 due to their distant similarity with secretin receptors [19]. Only two adhesion receptors could be detected in Fugu from the sequence dataset collected. Both corresponded to the Ig-hepta/GPR116 that is found in the chicken and human datasets of GPCRs, while 29 receptors were grouped in to this family for *Tetraodon nigroviridis*. No GPCRs corresponding to the BAI, CELSR, EMR, ETL, HE6 and LEC sub-families were found for Fugu in the public databases.

CD97 belongs to the B family of G protein-coupled receptors (GCPRs). Subfamily B2 contains cell surface molecules with long extracellular N-termini (LNB-TM7). They are putative cell-surface signaling molecules induced in the activated leukocytes and are highly expressed in regions of inflammation (indicating a probable protective and destructive immune responses), besides being expressed in smooth muscle cells and malignant tumors [20-22]. These receptors are present in *Tetraodon* and human but lack orthologs in Fugu and chicken, and were probably lost in the lineage leading to chicken.

### Frizzled/taste 2 (10 receptors)

The Frizzled/taste 2 sub-family are a comparatively recent addition to the GPCR family, which mediate signals from the secreted glycoproteins, thus controlling cell fate, polarity and proliferation during metazoan development [12]. They are receptors having 200 amino acid long N-termini with conserved cysteine residues that are expressed in the tongue and palate epithelium and probably act as bitter taste receptors, though their function is not quite clear.

This sub-family is one of the most highly-conserved among all GPCRs found in flies, fish and mammals, indicating an evolutionary pattern far-removed from the other GPCR families. In comparison to the 10 frizzled receptors of Fugu, the human GPCR repertoire has 24, while the count reaches 11 in the chicken sub-set. *Tetraodon* has been reported to have 12 frizzled/ smoothened/ TAS2 GPCRs.

The taste 2 GPCRs, which seem to have arisen much later, being conspicuously absent in flies, roundworm (*Caenorhabditis elegans*) and Fugu (*Takifugu rubripes*), have a considerable representation in the mouse and human GPCR sub-sets and a sole member in zebra-fish. In fact, this is one of the only two receptor types (the other being vomeronasal receptors) that have arisen after the split of tunicates from the lineage leading to the vertebrates. This receptor-type is not well-conserved; in fact they are among those that have evolved most rapidly in the past 100-200 million years in mammals.

### Secretin (24 receptors)

Secretin is one of the sub-families (besides Adhesion) that appeared on the GRAFS classification system by the splitting of class B of the A-F system. The secretins were found to be descendents of the adhesion family [23] and has structural similarity with the latter in their trans-membrane regions. The secretin receptors comprise of at least six highly conserved cysteine bridges in their N-termini and bind to large peptide ligands like hormones and neuropeptides [24]. They are well represented in all vertebrates as well as in tunicates, fruitfly and nematodes. They are gastro-intestinal hormones that regulate the ion (bicarbonate and potassium) and enzyme secretion from the pancreas. In Fugu, the secretin sub-family comprises of 2 calcitonin receptors (CALCR), 1 corticotropin-releasing hormone receptor (CRHR), 1 growth hormone-releasing hormone (GHRHR), 6 pituitary adeylate cyclase-activating peptide (PACAP), 6 parathyroid hormone receptors (PTHR) and 8 vasoactive intestinal peptide receptors (VIPR), bringing it to a total of 24, while in human and chicken there are 15 and 14 of these GPCRs, respectively. *Tetraodon* has 21 secretin receptors with at least 73% sequence similarity with the Fugu secretin receptors. The secretin and the VIPRs are thought to share a common ancestor, with secretin receptors appearing approximately 310 million years ago either due to gene duplication or from a glucagon ligand.

### Rhodopsins (56 olfactory, 148 non-olfactory)

**Figure 3:**
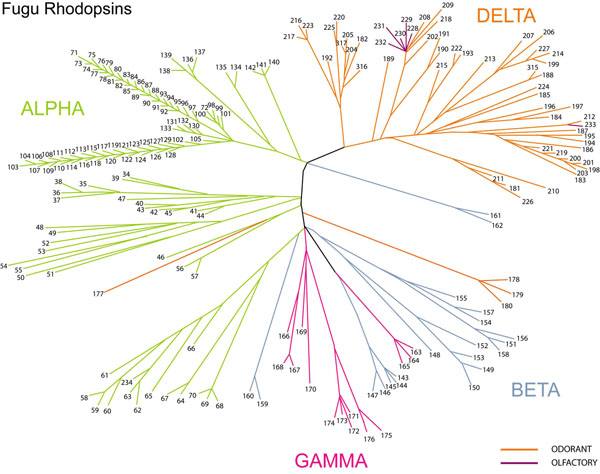
**Phylogenetic relationship between the receptors of the rhodopsin family in Fugu.** The figure depicts the members of the Fugu Rhodopsin family. The branches of this tree have been colored according to the four major sub-divisions in the family, namely- α, β, γ and δ receptor classes**.** The tree was constructed using the neighbor joining (NJ) method on the 1000 replicas of the 204 Rhodopsin receptor dataset. The numbering of the members is mentioned in the Dataset S3.

The Rhodopsins constitute the largest chunk (~64.5%) of the Fugu GPCR repertoire, comprising of 204 receptors, keeping with the trend in numbers seen in the GPCR repertoires of all vertebrates. Rhodopsins in the GRAFS system correspond to the class A of the A-F classification system. This is the first family of GPCRs whose three dimensional structure was determined, thus serving as the foundation of structural studies for the understanding of these special proteins. The rhodopsins have a structure that is different from adhesion, frizzled, secretin and majority of the glutamate receptors, as the rhodopsins have short N-termini, unlike the others mentioned. The rhodopsin binding cavity seems to be between the trans-membrane regions in contrast to the other GPCR families where the N-terminal plays the pivotal role in ligand-binding, though the ligand-binding domain of luteinizing hormone (LH), follicle stimulating hormone (FSH), leucine-rich-repeat containing GPCR (LG) and thyrotropin stimulating hormone (TSH) receptors of the δ sub-family lie in the N-termini [12] (Figure 3).

The Rhodopsin family is systematically categorized in to 4 groups based on experimental phylogenetic investigations: α(amine-binding receptors), β(only peptide-binding receptors), γ(receptors that bind to neuropeptides like somatostatins, galanin, opioids and chemokines etc.) and δ(olfactory, purine and glycoprotein receptors)[12].

### α–rhodopsins (110 receptors)

The α-group includes 24 amine receptors, 14 opsins and 72 members of the MECA cluster in the Fugu GPCR dataset. There are a total of 701 α-rhodopsins in human while 92 of these are present in chicken and 137 in *Tetraodon nigroviridis*. Some α-rhodopsin sub-groups like the melanocortin (MC) and endothelial differentiation G-protein coupled receptors (EDGRs) are very well-conserved. All but 3 out of the 63 Fugu MC receptors have one-to-one orthology with the human and chicken sub-sets, and both the EDGR receptors found exact matches in both human and chicken. The MC receptors (MC4 and MC5) arose early in the evolution of vertebrates, still displaying a remarkable conservation in their sequences, especially in the binding and activation domains [25]. Except the MC3 receptors, which Fugu lacks, its MC receptors have evident similarities with those found in mammals, especially in their pharmacological characters which indicates that they bind to melanocortin peptides with high potency and respond well in their presence, thus playing essential roles in pigmentation regulation, energy homeostasis and the production of steroids [26-28]. Fugu also lacks both prostaglandin and melatonin receptors of the α-subfamily but has 6 receptors each in the serotonin and dopamine subgroups, 1 muscarinic, 2 trace amines and 9 adrenergic receptors, all displaying one-to-one orthology with the human and chicken GPCRs. Fugu contains a total of 14 opsins: 5 rod pigments, 6 cone visual pigments, 3 encephalopsins/TMT (Teleost Multiple Tissue) opsins and no peropsins, melanopsins and retinal G-protein receptors. The MECA (MC, EDGR, Cannabinoid, ADORA/Adenosine receptor) cluster contains the highest number (72) of receptors in the rhodopsin family, out of which the majority (63) are MC receptors.

### β-rhodopsins (20 receptors)

Though this group has no main branches, it consists of 20 peptide-binding receptors of 17 different types. In the Fugu GPCR repertoire, this family is constituted by 10 neuropeptide Y (NPY), 5 neuropeptide (NPFF), 2 endothelin-related receptors (EDNR), 2 arginine-vasopressin receptors (AVPR) and 1 tachykinin receptors (TACR), but no hypocretin receptors (HCRTRs), cholecystokinin receptors (CCKs), gastrin-releasing peptide receptors (GRPRs), neuromedin B receptors (NMBRs), uterinbombesin receptors (BRS3), neurotensin receptors (NTSRs), growth hormone secretagogues receptor (GHSRs), neuromedin receptors (NMURs), thyrotropin releasing hormone receptors (TRHRs), ghrelins, gonadotropin releasing hormone receptors (GNRHRs), oxytocins or orphans. The human and chicken GPCR sub-sets have 35 and 45 β-rhodopsin receptors respectively, as compared to the 20 detected in Fugu and 88 in *Tetraodon nigroviridis*. All of the 10 Fugu NPY receptors show orthology with the human and chicken NPY receptors. Fugu has representatives of Y2, Y4 (also known as pancreatic polypeptide receptors or PP-receptors), Y7, Y8 and YY receptors types in this sub-class. The Y1, Y5, Y6 and the proposed Y3 receptors that are found in mammalian GPCR repertoires were not detected in the Fugu data-set. This sub-class gets its name from the fact that all NPY receptors bind with high affinity to the NPY and PYY peptides (with the exception of the Y4 receptors which prefer the peptide PP for binding).

### γ-rhodopsins (14 receptors)

This classification contains receptors that bind to both peptides and lipid-like compounds. The γ-rhodopsins of Fugu comprise of 14 receptors (in comparison with the 42 *Tetraodon nigroviridis*, 59 human and 46 chicken receptors that are placed in this category of rhodopsins) and are divided in to three main branches, namely- SOG (somatostatin, opioid and the neuropeptide galanin and RF-amide receptors), Melanin-concentrating hormone (MCH) and the chemokine receptor clusters. In Fugu, 6 SOG were detected out which 3 were classified as Neuropeptide galanin & the RF-amide binding receptor GALR and the remaining 3 as Somatostatin receptors (SSTRs 2 and 5). Opioids, which are receptors that are targeted for treating cough, pain and alcoholism, were not detected in Fugu. Two MCH receptors were detected in the Fugu GPCR repertoire, with representatives in both the MCH1 and MCH2 types, as in the case seen in mammals, though the latter seems to have been lost in chicken. The chemokine receptor cluster is an interesting group in this sub-class pertaining to their role in acute and chronic inflammation. Only one drug (for HIV treatment) has received regulatory approval that targets a chemokine called CCR5, which are used as co-receptors by some HIV strains during viral entry. In Fugu, 6 classic chemokines have been detected that comprise interleukin 8 receptors type I and II.

### δ-rhodopsins (60 receptors)

The final group of the rhodopsin sub-family is the δ-rhodopsins that have four main branches of MAS-related, glycoprotein, purine and the olfactory receptors clusters. In the Fugu GPCR dataset, the olfactory receptors form the major chunk of this sub-class, including 56 out of the 60 total δ-rhodopsins, in comparison to the 75, 518 and 290 such receptors found in *Tetraodon nigroviridis*, human and chicken, respectively. The remaining four receptors are representatives of the LHCGR (1 member) and the 3 members of the Relaxin-binding receptor sub-groups. The classic glycoprotein receptor hormones of LHCGR, FSHR and the TSHRs are members of the glycoprotein receptor sub-group which have been targets of recombinant peptides in different GPCR studies [29].Out of the 56 olfactory receptors in Fugu, 50 belong to the Odorant receptors, while the human genome has 388 functional odorant receptors [30,31], 229 such representatives are found in chicken and 23 odorant candidate receptors in the *Tetraodon nigroviridis* GPCR repertoire. Other than the same10 odorant receptors of the Fugu δ-rhodopsins, all other members were orthologous to the human and GPCR sub-sets. In fact, these were the only 10 receptors which did not have any human orthologs. The orphan GPCRs in *Tetraodon nigroviridis* could not be compared to those in Fugu as they were not grouped under any sub-families, unlike the latter. The high variability in the number of odorant genes in pisces indicates species-specific adaptation suited to bind to important receptors important to the specific species [11]. Six Fugu olfactory receptors could not be classified under any sub-category and hence, were placed under Other Olfactory Receptors.

The rapidly evolving class of olfactory GPCRs express themselves in the olfactory epithelium, with the help of which pisces, aves and mammals are known to detect the chemicals in their external environment. Olfactory receptors, on the basis of phylogenetic criteria [30,31], can broadly be categorized as those which recognize water-soluble odorants (Class I, which has been detected in both teleosts, including Fugu, and mammals, but lacking in chicken), and those that mediate air-borne smells (Class II, present in chicken and mammals, but not found in Fugu). Fishes use their olfactory system to detect pheromones for discriminating between toxins, predators, prey and mates, foraging, detecting nests and staying with their own school. Since majority of fishes lead a totally aquatic life, without ever leaving the water, they have no use for the Class II olfactory receptors. An olfactory receptor may bind to a diverse set of odorants and bring about activation. This may be the reason that as compared to fishes, mammals, especially humans, have an enormous number of odorant receptors of both the Class I and Class II types to enable them to discriminate between a plethora of smells. The characterization of these receptors would be of great impact and could be capitalized upon by the fragrance industry, though not so much as drug targets as they are yet to be implicated in the occurrence of any disease.

The two pufferfishes, Fugu and *Tetraodon nigroviridis*, have coding regions with approximately 87% similarity and both are considered model systems for studying human GPCRs [32]. Fugu and *Tetraodon nigroviridis* are separated by 18 to 30 million years that might add to the differences in their gene sequences. On account of its small size, availability and easier maintenance conditions, *Tetraodon nigroviridis* was the species of choice over Fugu initially. But following the first draft of the Fugu genome, revealing the huge similarity observed in three-fourth of Fugu proteins with that of humans, the comparability of their gene repertoire (as well as that of other vertebrates) was established and Fugu was accepted as a model system. The differences between the two pufferfishes might be due the fact that the two fishes differ in their natural habitat and thus have different environmental conditions to adapt to. But the study of both these systems adds to the annotation of both these species and is non-redundant.

## Conclusions

The phylogenetic analysis of GPCRs in the model system of Fugu helps to establish the position of fishes in the vertebrate evolution. The Fugu genome is one of the smallest vertebrate genomes that lack the repetitive sequences observed in genomes of higher organisms. It serves as a valuable reference set for studying other protein families in the human genome. We believe that a thorough understanding of the similarities and uniqueness of GPCRs among different genomes is vital before using them as drug targets. Moreover, such evolutionary studies with representatives of key vertebrate classes will help in identifying prime functional residues and motifs to elucidate the role of GPCRs in vertebrate evolution.

## Methods

### Sequence retrieval of Fugu GPCRs

Sequence database search was carried out on the *Takifugu rubripes* genome using ‘G Protein Coupled Receptors in *Takifugu rubripes*’ as the keywords. The sequence databases were re-checked with the individual receptor-types for more sequences. The sequences were extracted in the FASTA format using the Entrez search engine of National Center for Biotechnology Information (NCBI) [13], and Text Search of the Universal Protein Resource (UniProtKB) [14], which are search engines of the two non-redundant, publicly available databases and also from previously published literature [2]. The accession numbers for all the FASTA sequences can be found in the Additional File 1. The sequences retrieved were cross-checked using the NCBI Conserved Domain Database [33] for the 7TM conserved domain, which is a hall-mark of GPCRs.

### Classification and ortholog detection

The initial data set was classified using the IUPHAR-Database [34], PRED-GPCR database [6], BIAS-PROFS (Bioinformatics, Immunology and Algorithms make Short work of PROtein Function classification) [35], GPCRDB (Information System for G Protein-Coupled Receptors) [36] and references from previously published papers [1,3,12,16,24,28,37-46] (Additional File 1).

We detected orthologous relationships between genes of Fugu, human and chicken using systematic similarity searches at the protein level. Taking the dataset of the classified receptors, we compared it with the human and chicken genomes using NCBI Protein BLAST [47] with an extremely stringent similarity expect threshold of 1x10^-6^ for orthology detection [48].

### Phylogenetic analysis

The Fugu GPCRs were broadly classified into Non-Rhodopsins and Rhodopsins and their respective sub-families. The sequences of all the proteins (in FASTA format) were then combined into one file for performing multiple sequence alignment employing the JTT200 protein weight matrix in the MAFFT version 6.624 [49], using E-INS-I with a gap opening penalty of 1.53 and default offset value. SEQBOOT from the PHYLIP package [version 3.68] [49] was used on the ensuing datasets (i.e. the multiple sequence alignment files) and they were bootstrapped 1000 times. Subsequently 1000 (JTT) distance matrices were obtained using PROTDIST. The neighbor-joining method was employed through the NEIGHBOR program and CONSENSE generated a consensus of 1000 neighbour trees. Two additional multiple sequence alignment files comprising of Rhodopsins and the Non-Rhodopsins were also prepared, respectively, to get separate phylogenetic trees (their corresponding numbering in the tree has been mentioned in the Additional File 2) keeping the same parameters. The sequences were grouped based on the family identity observed with the human and chicken orthologs (Additional File 3). Sequences were categorized as ‘Other 7TM’ if they did not match the properties of any family of the GRAFS classification system.

## List of abbreviations used

GPCRs: G protein-coupled receptors; TM: trans-membrane; MC: melanocortin; GRAFS: Glutamate, Rhodopsin, Adhesion, Frizzled/TAS2, Secretin.

## Authors’ contributions

Corresponding author DS conceived the project; DS and AS designed the method and framework for the project. AS and SK performed the experiments and analyzed the data. DS, AS and SK wrote the manuscript.

## Competing interests

The authors declare that they have no competing interests.

## Supplementary Material

Additional File 1 Table of Classification of the 316 GPCR receptors of Fugu according to the GRAFS classification system. Click here for file

Additional File 2 Table enlisting all the Fugu GPCR sequences in the dataset and their corresponding names and numbering used in phylogenetic study using PHYLIP (version 3.68). Click here for file

Additional File 3 Table enlisting the orthologs of Fugu GPCRs in the human, chicken and *Tetraodon nigroviridis* GPCR sub-sets. Click here for file
