# Supplementary material for: The G protein-coupled receptors in the pufferfish Takifugu rubripes
Source: BMC Bioinformatics. 2011 Feb 15;12(Suppl 1):S3. doi: 10.1186/1471-2105-12-S1-S3 (PMC3044285; doi:10.1186/1471-2105-12-S1-S3)
Supplement: Additional File 1 — Table of Classification of the 316 GPCR receptors of Fugu according to the GRAFS classification system. [file 1471-2105-12-S1-S3-S1.pdf]

The G Protein-Coupled Receptors in the pufferfish *Takifugu rubripes*

Anita Sarkar[#], Sonu Kumar[#] and Durai Sundar[*]

Department of Biochemical Engineering and Biotechnology, Indian Institute of Technology (IIT) Delhi, New Delhi, India

Supporting Information

Additional File 1. Table of Classification of the 316 GPCR receptors of Fugu.

GLUTAMATE	
**1.**	**METABOTROPIC GLUTAMATE RECEPTORS (GRM)**
1)	O73641 (Metabotropic glutamate receptor 1 homologue (Fragment)) (UniProt unreviewed)
2)	O73642 (Metabotropic glutamate receptor 2 homologue (Fragment)) (UniProt unreviewed)
3)	O73643 (Metabotropic glutamate receptor 7 homologue (Fragment)) (UniProt unreviewed)
4)	O73644 (Metabotropic glutamate receptor 8 homologue (Fragment)) (UniProt unreviewed)
5)	BAA26128 (metabotropic glutamate receptor 1 homologue)
6)	BAA26129 (metabotropic glutamate receptor 2 homologue)
7)	BAA26130 (metabotropic glutamate receptor 7 homologue)
8)	BAA26131 (metabotropic glutamate receptor 8 homologue)
9)	CAA09083 (metabotropic glutamate receptor 7)
**2.**	**GABA RECEPTORS**
**3.**	**CALCIUM-SENSING RECEPTOR (CASR)**

1)	O73635 (Calcium2+ sensing receptor)(UniProt unreviewed)
**4.**	**TASTE RECEPTORS (TAS 1)**
1)	BAE78486 (taste receptor, type 1, member 1)
2)	NP_001091094 (taste receptor, type 1, member 1)
3)	Q2MHK1 (Taste receptor, type 1, member 1) (UniProt unreviewed)
4)	BAE78487 (taste receptor, type 1, member 2a)
5)	NP_001098687 (taste receptor, type 1, member 2a)
6)	Q2MHK0 (Taste receptor, type 1, member 2a) (UniProt unreviewed)
7)	BAE78488 (taste receptor, type 1, member 2b)
8)	NP_001098688 (taste receptor, type 1, member 2b)
9)	Q2MHJ9 (Taste receptor, type 1, member 2b) (UniProt unreviewed)
10)	BAE78489 (taste receptor, type 1, member 3)
11)	NP_001072097 (taste receptor, type 1, member 3)
12)	Q2MHJ8 (Taste receptor, type 1, member 3) (UniProt unreviewed)
**5.**	**PHEROMONE RECEPTORS (V2R) (ABSENT IN HUMANS)**
1)	BAA26123 (pheromone receptor)
2)	BAA26124 (pheromone receptor)
3)	BAA26125 (pheromone receptor)
4)	BAA26126 (pheromone receptor)
5)	BAA26127 (pheromone receptor)
6)	BAA26134 (pheromone receptor)
7)	BAA26135 (pheromone receptor)
8)	BAA26136 (pheromone receptor)
9)	BAA26137 (pheromone receptor)
10)	BAA26138 (pheromone receptor)

11)	BAA26139 (pheromone receptor)
12)	BAA26140 (pheromone receptor)
13)	BAA26141 (pheromone receptor)
14)	BAA26142 (pheromone receptor)
15)	BAA26143 (pheromone receptor)
16)	BAA26144 (pheromone receptor)
17)	BAA26145 (pheromone receptor)
18)	BAA26146 (pheromone receptor)
19)	O73636 (Pheromone receptor) (UniProt unreviewed)
20)	O73637 (Pheromone receptor) (UniProt unreviewed)
21)	O73638 (Pheromone receptor) (UniProt unreviewed)
22)	O73639 (Pheromone receptor) (UniProt unreviewed)
23)	O73640 (Pheromone receptor) (UniProt unreviewed)
24)	O73645 (Pheromone receptor (Fragment)) (UniProt unreviewed)
25)	O73646 (Pheromone receptor (Fragment)) (UniProt unreviewed)
26)	O73647 (Pheromone receptor (Fragment)) (UniProt unreviewed)
27)	O73648 (Pheromone receptor (Fragment)) (UniProt unreviewed)
28)	O73649 (Pheromone receptor (Fragment)) (UniProt unreviewed)
29)	O73650 (Pheromone receptor (Fragment)) (UniProt unreviewed)
30)	O73651 (Pheromone receptor (Fragment)) (UniProt unreviewed)
31)	O73652 (Pheromone receptor (Fragment)) (UniProt unreviewed)
32)	O73653 (Pheromone receptor (Fragment)) (UniProt unreviewed)
33)	O73654 (Pheromone receptor (Fragment)) (UniProt unreviewed)
34)	O73655 (Pheromone receptor (Fragment)) (UniProt unreviewed)
35)	O73656 (Pheromone receptor (Fragment)) (UniProt unreviewed)

36)	O73657 (Pheromone receptor (Fragment)) (UniProt unreviewed)

RHODOPSIN	
S. No	ALPHA (5)
**1**	**PROSTAGLANDIN RECEPTOR CLUSTER (2)**
i.	Prostaglandin receptors
ii.	Orphan Prostaglandin receptors
**2**	**AMINE RECEPTOR CLUSTER (7)**
i.	Serotonin receptors (HTR)
1)	CAA58745
2)	CAA65175
3)	CAA65176
4)	O42385 (Serotonin receptor 1A-α)
5)	O42384 (Serotonin receptor 1A-β)
6)	P79748 (Serotonin receptor 1D)
ii.	Dopamine receptors (DRD)
1)	CAA56455
2)	CAA56456
3)	CAA56457
4)	P53452 (Dopamine receptor)
5)	P53453 (Dopamine receptor)
6)	P53454 (Dopamine receptor)

iii.	Muscarinic receptors (CHRM)
1)	AAU09270 (M2)
iv.	Histamine receptors (HRH)
v.	Adrenergic receptors (ADR)
1)<br>2)<br>3)<br>4)<br>5)<br>6)<br>7)<br>8)<br>9)	AAQ02695 (putative β-2)<br>CAC87880 (α2 a1)<br>CAC87884 (α2 a2)<br>CAC87882 (α2 b1)<br>CAC87883 (α2 b2)<br>CAC87881 (α2 c1)<br>CAC87885 (α2 c2)<br>CAC87886 (α2 d1)<br>CAC87887 (α2 d2)
vi.	Trace Amine receptors (TAR)
1)<br>2)	AAC96117 (putative neurotransmitter receptor)<br>AAC96118 (putative neurotransmitter receptor)
vii.	Orphan amine cluster receptors
**3**	**OPSINS RECEPTOR CLUSTER (6)**
i.	Rod visual pigment (RHO)
1)<br>2)<br>3)<br>4)	AAD54580 (rhodopsin)<br>AAF44621<br>AAF44622<br>NP_001029021 (rod-like opsin)

5)	NP_001072099
ii.	Cone visual pigment (OPN etc.)
1)<br>2)<br>3)<br>4)<br>5)<br>6)	AAF44648 (green opsin)<br>NP_001028884 (green opsin)<br>Q7T2L3 (GPR136 →confirm/undetermined) (UniProt unreviewed)<br>AAT38456 (red-sensitive/ iodopsin)<br>AAT38459 (blue-sensitive)<br>AAP72121 (GPR136 →confirm/undetermined)
iii.	Peropsin (RRH)
iv.	Encephalopsin (/**T**eleost **M**ultiple **T**issue Opsin)
1)<br>2)<br>3)	AAL83430<br>AAM90677<br>NP_001027778
v.	Melanopsin
vi.	Retinal G-protein coupled receptor (RGR)
**4**	**MELATONIN RECEPTOR CLUSTER (2)**
i.	Melatonin receptors
ii.	Orphan melatonin cluster receptor
**5**	**MECA RECEPTOR CLUSTER (5)**

i.	Melanocortin (MC) receptors
1)	AAO24749 (MC 1)
2)	AAO65548 (MC 1)
3)	BAG38415 (MC 1)
4)	BAG38416 (MC 1)
5)	BAG38417 (MC 1)
6)	BAG38418 (MC 1)
7)	BAG38419 (MC 1)
8)	BAG38420 (MC 1)
9)	BAG38421 (MC 1)
10)	BAG38422 (MC 1)
11)	BAG38423 (MC 1)
12)	BAG38424 (MC 1)
13)	BAG38425 (MC 1)
14)	BAG38426 (MC 1)
15)	BAG38427 (MC 1)
16)	BAG38428 (MC 1)
17)	BAG38429 (MC 1)
18)	BAG38430 (MC 1)
19)	BAG38431 (MC 1)
20)	BAG38432 (MC 1)
21)	BAG38433 (MC 1)
22)	BAG38434 (MC 1)
23)	BAG38435 (MC 1)
24)	BAG38436 (MC 1)

25)	BAG38437 (MC 1)
26)	BAG38438 (MC 1)
27)	NP_001127927 (MC 1)
28)	AAO24748 (MC 2)
29)	AAO65549 (MC 2)
30)	AAO65550 (MC 2)
31)	NP_001027936 (MC 2)
32)	AAO24751 (MC 4)
33)	AAO65551 (MC 4)
34)	BAB71730 (MC 4)
35)	BAG38469 (MC 4)
36)	BAG38470 (MC 4)
37)	BAG38471 (MC 4)
38)	BAG38472 (MC 4)
39)	BAG38473 (MC 4)
40)	BAG38474 (MC 4)
41)	BAG38475 (MC 4)
42)	BAG38476 (MC 4)
43)	BAG38477 (MC 4)
44)	BAG38478 (MC 4)
45)	BAG38479 (MC 4)
46)	BAG38480 (MC 4)
47)	BAG38481 (MC 4)
48)	BAG38482 (MC 4)
49)	BAG38483 (MC 4)

50)	BAG38484 (MC 4)
51)	BAG38485 (MC 4)
52)	BAG38486 (MC 4)
53)	BAG38487 (MC 4)
54)	BAG38488 (MC 4)
55)	BAG38489 (MC 4)
56)	BAG38490 (MC 4)
57)	BAG38491 (MC 4)
58)	BAG38492 (MC 4)
59)	NP_001027732 (MC 4)
60)	AAO24750 (MC 5)
61)	AAO65552 (MC 5)
62)	AAO65553 (MC 5)
63)	NP_001027937 (MC 5)
ii.	Endothelial Differentiation G-protein coupled receptors (EDGR)
1)	Q9PUQ8 (Sphingosine 1-phosphate receptor Edg-3)
2)	AAF07896 (EDG-3)
iii.	Cannabinoid receptor (CNR)
1)	CAA64174 (Type 1A)
2)	Q98894 (Type 1A)
3)	CAA64175 (Type 1B)
4)	Q98895 (Type 1B)
iv.	Adenosine binding receptors (ADORA)
v.	Orphan MECA cluster receptors

1)<br><br>2)<br><br>3)	AAP72120 (GPR119)<br><br>NP_001027835 (GPR119)<br><br>Q7T2L4 (GPR119) (UniProt unreviewed)
S. No.	BETA (has no main branches)
1	Hypocretin receptors (HCRTR)
2	Neuropeptide FF (NPFF) receptors
1)<br><br>2)<br><br>3)<br><br>4)<br><br>5)	BAF34888 (neuropeptide FF receptor-1 NPFF2-1)<br><br>NP_001092118 (neuropeptide FF receptor-1 NPFF2-1)<br><br>Q05KN3 (neuropeptide FF receptor-1 NPFF2-1) (UniProt unreviewed)<br><br>BAF34889 (neuropeptide FF receptor-2 NPFF2-2)<br><br>NP_001092119 (neuropeptide FF receptor-2 NPFF2-2)
3	Tachykinin receptors (TACR)
1)	AAQ02694 (putative tachykinin receptor 1)
4	Cholecystokinin (CCK) receptors
5	Neuropeptide Y (NPY) receptors
1)<br><br>2)<br><br>3)<br><br>4)<br><br>5)<br><br>6)<br><br>7)<br><br>8)	ABU87345 (NPY2R)<br><br>NP_001098693 (NPY receptor2)<br><br>ABU87346 (NPY4R)<br><br>ABU87347 (NPY7R)<br><br>NP_001098695 (neuropeptide Y receptor Y7)<br><br>ABU87348 (NPY8aR)<br><br>ABU87349 (NPY8bR)<br><br>NP_001098073 (neuropeptide Y/peptide YY receptor)

9)<br><br>10)	NP_001098074 (neuropeptide Y/peptide YY receptor)<br><br>NP_001098075 (neuropeptide Y/peptide YY receptor)
6	Endothelin-related receptors (EDNR & ETBRLP1/2)
1)<br><br>2)	BAF47415 (Type A)<br><br>NP_001092135 (Type A)
7	Gastrin-releasing peptide receptor (GRPR)
8	Neuromedin B receptor (NMBR)
9	Uterinbombesin receptor (BRS3)
10	Neurotensin receptors (NTSR)
11	Growth hormone secretagogues receptor (GHSR)
12	Neuromedin receptors (NMUR)
13	Thyrotropin releasing hormone receptor (TRHR)
14	Ghrelin receptor
15	Arginine Vasopressin receptors (AVPR)
1)<br><br>2)	AAK17004<br><br>AAK18744
16	Gonadotropin-releasing hormone receptors (GNRHR)

17	Oxytocin receptor (OXTR)
18	Orphan beta group receptors
S. No.	GAMMA (3)
1	SOG RECEPTOR CLUSTER (3)
i.	Neuropeptide galanin & the RF-amide binding receptor GALR
1)<br><br>2)<br><br>3)	BAF34887 (RFamide-related peptide receptor)<br><br>Q05KN4 (RFamide-related peptide receptor) (UniProt unreviewed)<br><br>NP_001092117 (RFamide-related peptide receptor)
ii.	Somatostatin receptors (SSTR)
1)<br><br>2)<br><br>3)	AAL32173<br><br>O42179 (Somatostatin-like receptor)<br><br>AAB86684
iii.	Opioid receptors (OPR)
**2**	MELANIN-CONCENTRATING HORMONE (MCH) RECEPTOR (0)
1)<br><br>2)	AAO24755 (MCH 1)<br><br>AAO24756 (MCH 2)
**3**	CHEMOKINE RECEPTOR CLUSTER (3)
i.	Classic chemokines (CCRs, CXCRs)
1)<br><br>2)	AAY41945 (interleukin 8 receptor I transcript 1)<br><br>NP_001072110 (interleukin 8 receptor I transcript 1)

3)	AAY41946 (interleukin 8 receptor I transcript 2)
4)	NP_001091093 (interleukin 8 receptor I transcript 2)
5)	AAY41947 (interleukin 8 receptor II)
6)	NP_001072090 (interleukin 8 receptor II)
ii.	Angiotensin (AGTR)/ bradykinin (BDKRB)-related receptors
iii.	Orphan chemokine receptor
S. No.	DELTA (4)
**1**	MAS-RELATED RECEPTOR CLUSTER (2)
i.	MAS1 oncogene receptor (MAS)
ii.	MAS-related receptor (MRG & MRGX)
**2**	GLYCOPROTEIN RECEPTOR CLUSTER (2)
i.	Classic glycoprotein hormone receptors (3)
a.	FSHR
b.	TSHR
c.	LHCGR
1)	DAA06178 (TPA_inf: luteinizing hormone receptor)
ii.	Leucine-rich-repeat containing G-protein coupled receptors (LGR) (3)
a.	Relaxin-binding receptors (LGR7-8)

1)<br><br>2)<br><br>3)	AAP72119 (GPR100 → confirm/undetermined)<br><br>NP_001027859<br><br>Q7T2L5 (GPR100 →confirm/undetermined) (UniProt unreviewed)
b.	Orphan LGR4-6
c.	Glycoprotein hormone receptors
**3**	PURINE RECEPTOR CLUSTER(3)
i.	Formyl peptide receptors (FPR)
ii.	Nucleotide-binding & related receptors
a.	Nucleotide binding receptors (P2Y)
b.	Thrombin receptors (F2R)
c.	Cysteinyl leukotriene receptors (CYSLT)
d.	Orphan purine receptors
**4**	OLFACTORY RECEPTOR CLUSTER (17)
**i.**	Odorant receptor
1)<br><br>2)<br><br>3)<br><br>4)	ABC43425<br><br>ABC43426<br><br>ABC43427<br><br>ABC43428

5)	ABC43429
6)	ABC43430
7)	ABC43431
8)	ABC43432
9)	ABC43433
10)	ABC43434
11)	ABC43435
12)	ABC43436
13)	ABC43437
14)	ABC43438
15)	ABC43439
16)	ABC43440
17)	ABC43441
18)	ABC43442
19)	ABC43443
20)	ABC43444
21)	ABC43445
22)	ABC43446
23)	ABC43447
24)	ABC43448
25)	ABC43449
26)	ABC43450
27)	ABC43451
28)	ABC43452
29)	ABC43453

30)	ABC43454
31)	ABC43455
32)	ABC43456
33)	ABC43457
34)	ABC43458
35)	ABC43459
36)	ABC43460
37)	ABC43461
38)	ABC43462
39)	ABC43463
40)	ABC43464
41)	ABC43465
42)	ABC43466
43)	ABC43467
44)	ABC43468
45)	ABC43469
46)	ABC43470
47)	ABC43471
48)	ABC43472
49)	ABC43473
50)	ABC43474
**ii.**	**OTHER OLFACTORY RECEPTORS**
1)	BAA92165 (olfactory receptor 1-1)
2)	BAA92166 (olfactory receptor 1-2)
3)	BAA92167 (olfactory receptor 1-3)

4)	BAA92168 (olfactory receptor 1-4)
5)	BAA92169 (olfactory receptor 1-5)
6)	BAA92170 (olfactory receptor 2)

ADHESION	
**S.No.**	**GPR116**
1)	BAF32963 (flg-Hepta) / GPR116
2)	NP_001092114 (flg-Hepta protein) / GPR116

FRIZZLED	
**S. No.**	**(*from Fredrikkson et al.'s dataset)**
1.	scaffold_300.214923.216626
2.	scaffold_1549.10885.12627
3.	scaffold_536.20133.21884
4.	scaffold_2461.12412.14142
5.	scaffold_74.265266.266879
6.	scaffold_1909.3949.8009
7.	scaffold_43.402294.403766
8.	scaffold_6516.7.1682
9.	scaffold_849.34084.38418
10.	scaffold_3606.3197.10767

SECRETIN	
**1.**	**CALCITONIN RECEPTOR (CALCR)**
1)	FAA00372 (TPA: calcitonin receptor)

2)	NP_001098689
**2.**	**CORTICOTROPIN-RELEASING HORMONE RECEPTORS (CRHR)**
1)	CAC82924 (corticotrophin releasing factor receptor)
**3.**	**GLUCAGON RECEPTOR (GCGR)**
**4.**	**GASTRIC INHIBITORY POLYPEPTIDE RECEPTOR (GIPR)**
**5.**	**GLUCAGON-LIKE PEPTIDE RECEPTORS**
**6.**	**GROWTH HORMONE-RELEASING HORMONE RECEPTOR (GHRHR)**
1)	CAC82589 (growth hormone releasing hormone-like)
**7.**	**PITUITARY ADENYLATE CYCLASE-ACTIVATING PEPTIDE (PACAP)**
1)<br>2)<br>3)<br>4)<br>5)<br>6)	CAD35690 (pituitary adenylate-cyclase activating polypeptide receptor 1A)<br>CAD38842 (pituitary adenylate cyclase-activating polypeptide 1B)<br>Q5WML0 (Pituitary adenylate cyclase-activating polypeptide 1B) (UniProt unreviewed)<br>Q5WML1 (Pituitary adenylate-cyclase activating polypeptide receptor 1A) (UniProt unreviewed)<br>NP_001098685 (pituitary adenylate-cyclase activating polypeptide receptor 1A)<br>NP_001098686 (pituitary adenylate cyclase-activating polypeptide 1B)
**8.**	**PARATHYROID HORMONE RECEPTORS (PTHR)**
1)<br>2)<br>3)<br>4)<br>5)	CAD67555 (parathyroid hormone receptor 3)<br>CAD68048<br>CAD79707 (parathyroid hormone receptor 1)<br>Q2UZQ9 (UniProt unreviewed)<br>Q2UZQ8 (Parathyroid hormone receptor 1) (UniProt unreviewed)

6)	Q2UZR0 (Parathyroid hormone receptor 3) (UniProt unreviewed)
**9.**	**SECRETIN RECEPTOR (SCTR)**
**10.**	**VASOACTIVE INTESTINAL PEPTIDE RECEPTOR (VIPR)**
1)	CAC82587 (vasoactive intestinal peptide receptor)
2)	CAC82588 (vasoactive intestinal peptide receptor 1 A)
3)	CAC83860 (vasoactive intestinal peptide receptor)
4)	CAC83861 (vasoactive intestinal peptide receptor)
5)	Q802T6 (Vasoactive intestinal peptide receptor (Fragment)) (vipr2B)
6)	Q802T7 (Vasoactive intestinal peptide receptor (Fragment)) (vipr2A)
7)	Q8AXV3 (Vasoactive intestinal peptide receptor 1 A) (vipr1A)
8)	Q8AXV4 (Vasoactive intestinal peptide receptor) (vipr1B)

OTHER 7TM RECEPTORS

S. No.	OTHER 7TM RECEPTORS
1)	AAP04328 (GPR34)
2)	AAP72122 (GPR142a)
3)	Q7T2L2 (GPR142a) (UniProt unreviewed)
4)	AAP72123 (GPR142b)
5)	Q7T2L1 (GPR142b) (Fragment) (UniProt unreviewed)
6)	AAP72142 (GPR135)
7)	NP_001027836 (GPR135)
8)	Q7T2L0 (GPR135) (UniProt unreviewed)
9)	ABL01522 (V1R pheromone receptor-like protein)

10)	NP_001098705 (GPR39-1)
11)	ABU53899 (GPR39-1a)
12)	ABU53900 (GPR39-1b)
13)	NP_001098703 (GPR155)
14)	ABF22455 (GPR155)
15)	ABF22483 (GPR155)
16)	ABD61705 (progestin and adipoQ receptor family member VII)
17)	NP_001035912 (progestin and adipoQ receptor family member VII)
18)	Q19WU5 (Progestin and adipoQ receptor family member VII (MPRA) (UniProt unreviewed))
