# Supplementary material for: The G protein-coupled receptors in the pufferfish Takifugu rubripes
Source: BMC Bioinformatics. 2011 Feb 15;12(Suppl 1):S3. doi: 10.1186/1471-2105-12-S1-S3 (PMC3044285; doi:10.1186/1471-2105-12-S1-S3)
Supplement: Additional File 2 — Table enlisting all the Fugu GPCR sequences in the dataset and their corresponding names and numbering used in phylogenetic study using PHYLIP (version 3.68). [file 1471-2105-12-S1-S3-S2.pdf]

The G Protein-Coupled Receptors in the pufferfish *Takifugu rubripes*

Anita Sarkar#, Sonu Kumar# and Durai Sundar*

Department of Biochemical Engineering and Biotechnology, Indian Institute of Technology (IIT) Delhi, New Delhi, India

Supporting Information

Additional File 2. Table enlisting all the Fugu GPCR sequences in the dataset and their corresponding names and numbering used in the phylogenetic study

Serial Number	Original Accession number	Name in the tree
1.	tr|O73641|O73641_FUGRU Metabotropic glutamate receptor 1 homologue (Fragment)	**G**|GRM_1
2.	tr|O73642|O73642_FUGRU Metabotropic glutamate receptor 2 homologue (Fragment)	**G**|GRM_2
3.	tr|O73643|O73643_FUGRU Metabotropic glutamate receptor 7 homologue (Fragment)	**G**|GRM_3
4.	tr|O73644|O73644_FUGRU Metabotropic glutamate receptor 8 homologue (Fragment)	**G**|GRM_4
5.	tr|O73635|O73635_FUGRU Calcium2+ sensing receptor	**G**|CASR_1
6.	tr|Q2MHK1|Q2MHK1_FUGRU Taste receptor, type 1, member 1	**G**|TAS1_1
7.	gi|85677369|dbj|BAE78487.1| taste receptor, type 1, member 2a	**G**|TAS1_2
8.	gi|157412288|ref|NP_001098687.1| taste receptor, type 1, member 2a	**G**|TAS1_3
9.	tr|Q2MHK0|Q2MHK0_FUGRU Taste receptor, type 1, member 2a	**G**|TAS1_4
10.	gi|85677371|dbj|BAE78488.1| taste receptor, type 1, member 2b	**G**|TAS1_5

11.	gi|157412290|ref|NP_001098688.1| taste receptor, type 1, member 2b	**G**|TAS1_6
12.	tr|Q2MHJ9|Q2MHJ9_FUGRU Taste receptor, type 1, member 2b	**G**|TAS1_7
13.	gi|85677373|dbj|BAE78489.1| taste receptor, type 1, member 3	**G**|TAS1 8
14.	gi|118344624|ref|NP_001072097.1| taste receptor, type 1, member 3	**G**|TAS1_9
15.	tr|Q2MHJ8|Q2MHJ8_FUGRU Taste receptor, type 1, member 3	**G**|TAS1_10
16.	tr|O73636|O73636_FUGRU Pheromone receptor	**G**|V2R_1
17.	tr|O73637|O73637_FUGRU Pheromone receptor	**G**|V2R_2
18.	tr|O73638|O73638_FUGRU Pheromone receptor	**G**|V2R_3
19.	tr|O73639|O73639_FUGRU Pheromone receptor	**G**|V2R_4
20.	tr|O73640|O73640_FUGRU Pheromone receptor	**G**|V2R_5
21.	tr|O73645|O73645_FUGRU Pheromone receptor (Fragment)	**G**|V2R_6
22.	tr|O73646|O73646_FUGRU Pheromone receptor (Fragment)	**G**|V2R_7
23.	tr|O73647|O73647_FUGRU Pheromone receptor (Fragment)	**G**|V2R_8
24.	tr|O73648|O73648_FUGRU Pheromone receptor (Fragment)	**G**|V2R_9
25.	tr|O73649|O73649_FUGRU Pheromone receptor (Fragment)	**G**|V2R_10
26.	tr|O73650|O73650_FUGRU Pheromone receptor (Fragment)	**G**|V2R_11
27.	tr|O73651|O73651_FUGRU Pheromone receptor (Fragment)	**G**|V2R_12

28.	tr|O73652|O73652_FUGRU Pheromone receptor (Fragment)	**G**|V2R_13
29.	tr|O73653|O73653_FUGRU Pheromone receptor (Fragment)	**G**|V2R_14
30.	tr|O73654|O73654_FUGRU Pheromone receptor (Fragment)	**G**|V2R_15
31.	tr|O73655|O73655_FUGRU Pheromone receptor (Fragment)	**G**|V2R_16
32.	tr|O73656|O73656_FUGRU Pheromone receptor (Fragment)	**G**|V2R_17
33.	tr|O73657|O73657_FUGRU Pheromone receptor (Fragment)	**G**|V2R_18
34.	gi|1770280|emb|CAA58745.1| serotonin receptor	HTR_1
35.	gi|2274951|emb|CAA65175.1| serotonin receptor	HTR_2
36.	gi|2274949|emb|CAA65176.1| serotonin receptor	HTR_3
37.	gi|6224982|sp|O42385.1|5H1AA_FUGRU RecName: Full=5-hydroxytryptamine receptor 1A-alpha	HTR_4
38.	gi|6224983|sp|O42384.1|5H1AB_FUGRU RecName: Full=5-hydroxytryptamine receptor 1A-beta	HTR_5
39.	gi|2494925|sp|P79748.1|5HT1D_FUGRU RecName: Full=5-hydroxytryptamine receptor 1D	HTR_6
40.	gi|1204090|emb|CAA56455.1| dopamine receptor	DRD_1
41.	gi|1204092|emb|CAA56456.1| dopamine receptor	DRD_2
42.	gi|1204095|emb|CAA56457.1| dopamine receptor	DRD_3
43.	gi|1706280|sp|P53452.1|DRD1L_FUGRU RecName: Full=D(1)-like dopamine receptor	DRD_4
44.	gi|1706284|sp|P53453.1|DRD2L_FUGRU RecName: Full=D(2)-like dopamine receptor	DRD_5

45.	gi|1706290|sp|P53454.1|DRD5L_FUGRU RecName: Full=D(5)-like dopamine receptor	DRD_6
46.	gi|51703258|gb|AAU09270.1| M2 muscarinic acetylcholine receptor	CHRM_1
47.	gi|33304630|gb|AAQ02695.1| putative beta-2 adrenergic receptor	ADR_1
48.	gi|21322658|emb|CAC87880.1| alpha2 a1 adrenergic receptor	ADR_2
49.	gi|21322666|emb|CAC87884.1| alpha2 a2 adrenergic receptor	ADR_3
50.	gi|21322662|emb|CAC87882.1| alpha2 b1 adrenergic receptor	ADR_4
51.	gi|21322664|emb|CAC87883.2| alpha2 b2 adrenergic receptor	ADR_5
52.	gi|21322660|emb|CAC87881.1| alpha2 c1 adrenergic receptor	ADR_6
53.	gi|21322668|emb|CAC87885.1| alpha2 c2 adrenergic receptor	ADR_7
54.	gi|21322670|emb|CAC87886.1| alpha2 d1 adrenergic receptor	ADR_8
55.	gi|21322672|emb|CAC87887.1| alpha2 d2 adrenergic receptor	ADR_9
56.	gi|4028153|gb|AAC96117.1| putative neurotransmitter receptor	TAR_1
57.	gi|4028154|gb|AAC96118.1| putative neurotransmitter receptor	TAR_2
58.	gi|7271783|gb|AAF44621.1|AF201471_1 rod opsin	RHO_1
59.	gi|7271785|gb|AAF44622.1|AF201472_1 rod-like opsin	RHO_2
60.	gi|76362826|ref|NP_001029021.1| rod-like opsin	RHO_3

61.	gi|118344636|ref|NP_001072099.1| rod opsin	RHO_4
62.	gi|7271838|gb|AAF44648.1|AF226989_1 green opsin	CONE_1
63.	gi|76253896|ref|NP_001028884.1| green opsin	CONE_2
64.	tr|Q7T2L3|Q7T2L3_FUGRU G protein-coupled receptor 136 (Fragment)	CONE_3
65.	gi|47779145|gb|AAT38456.1| red-sensitive pigment	CONE_4
66.	gi|47779153|gb|AAT38459.1| blue-sensitive pigment	CONE_5
67.	gi|32165508|gb|AAP72121.1| G protein-coupled receptor 136	CONE_6
68.	gi|19032632|gb|AAL83430.1|AF349945_1 TMT opsin	TMT_1
69.	gi|22086563|gb|AAM90677.1|AF402774_1 multiple tissue opsin	TMT_2
70.	gi|74095897|ref|NP_001027778.1| multiple tissue opsin	TMT_3
71.	gi|28827152|gb|AAO24749.1| melanocortin 1 receptor	MC_1
72.	gi|28827150|gb|AAO24748.1| melanocortin 2 receptor	MC_2
73.	gi|189068428|dbj|BAG38415.1| melanocortin 1 receptor	MC_3
74.	gi|189068430|dbj|BAG38416.1| melanocortin 1 receptor	MC_4
75.	gi|189068432|dbj|BAG38417.1| melanocortin 1 receptor	MC_5
76.	gi|189068434|dbj|BAG38418.1| melanocortin 1 receptor	MC_6

77.	gi|189068436|dbj|BAG38419.1| melanocortin 1 receptor	MC_7
78.	gi|189068438|dbj|BAG38420.1| melanocortin 1 receptor	MC_8
79.	gi|189068440|dbj|BAG38421.1| melanocortin 1 receptor	MC_9
80.	gi|189068442|dbj|BAG38422.1| melanocortin 1 receptor	MC_10
81.	gi|189068444|dbj|BAG38423.1| melanocortin 1 receptor	MC_11
82.	gi|189068446|dbj|BAG38424.1| melanocortin 1 receptor	MC_12
83.	gi|189068448|dbj|BAG38425.1| melanocortin 1 receptor	MC_13
84.	gi|189068450|dbj|BAG38426.1| melanocortin 1 receptor	MC_14
85.	gi|189068452|dbj|BAG38427.1| melanocortin 1 receptor	MC_15
86.	gi|189068454|dbj|BAG38428.1| melanocortin 1 receptor	MC_16
87.	gi|189068456|dbj|BAG38429.1| melanocortin 1 receptor	MC_17
88.	gi|189068458|dbj|BAG38430.1| melanocortin 1 receptor	MC_18
89.	gi|189068460|dbj|BAG38431.1| melanocortin 1 receptor	MC_19
90.	gi|189068462|dbj|BAG38432.1| melanocortin 1 receptor	MC_20
91.	gi|189068464|dbj|BAG38433.1| melanocortin 1 receptor	MC_21
92.	gi|189068466|dbj|BAG38434.1| melanocortin 1	MC_22

	receptor	
93.	gi|189068468|dbj|BAG38435.1| melanocortin 1 receptor	MC_23
94.	gi|189068470|dbj|BAG38436.1| melanocortin 1 receptor	MC_24
95.	gi|189068472|dbj|BAG38437.1| melanocortin 1 receptor	MC_25
96.	gi|189068474|dbj|BAG38438.1| melanocortin 1 receptor	MC_26
97.	gi|197333798|ref|NP_001127927.1| melanocortin 1 receptor	MC_27
98.	gi|28827150|gb|AAO24748.1| melanocortin 2 receptor	MC_28
99.	gi|29165376|gb|AAO65549.1| melanocortin 2 receptor	MC_29
100.	gi|29165378|gb|AAO65550.1| melanocortin 2 receptor	MC_30
101.	gi|74136155|ref|NP_001027936.1| melanocortin 2 receptor	MC_31
102.	gi|28827156|gb|AAO24751.1| melanocortin 4 receptor	MC_32
103.	gi|29165380|gb|AAO65551.1| melanocortin 4 receptor	MC_33
104.	gi|16580725|dbj|BAB71730.1| melanocortin receptor-4	MC_34
105.	gi|189069012|dbj|BAG38469.1| melanocortin 4 receptor	MC_35
106.	gi|189069014|dbj|BAG38470.1| melanocortin 4 receptor	MC_36
107.	gi|189069016|dbj|BAG38471.1| melanocortin 4 receptor	MC_37

108.	gi|189069018|dbj|BAG38472.1| melanocortin 4 receptor	MC_38
109.	gi|189069020|dbj|BAG38473.1| melanocortin 4 receptor	MC_39
110.	gi|189069022|dbj|BAG38474.1| melanocortin 4 receptor	MC_40
111.	gi|189069024|dbj|BAG38475.1| melanocortin 4 receptor	MC_41
112.	gi|189069026|dbj|BAG38476.1| melanocortin 4 receptor	MC_42
113.	gi|189069028|dbj|BAG38477.1| melanocortin 4 receptor	MC_43
114.	gi|189069030|dbj|BAG38478.1| melanocortin 4 receptor	MC_44
115.	gi|189069032|dbj|BAG38479.1| melanocortin 4 receptor	MC_45
116.	gi|189069034|dbj|BAG38480.1| melanocortin 4 receptor	MC_46
117.	gi|189069036|dbj|BAG38481.1| melanocortin 4 receptor	MC_47
118.	gi|189069038|dbj|BAG38482.1| melanocortin 4 receptor	MC_48
119.	gi|189069040|dbj|BAG38483.1| melanocortin 4 receptor	MC_49
120.	gi|189069042|dbj|BAG38484.1| melanocortin 4 receptor	MC_50
121.	gi|189069044|dbj|BAG38485.1| melanocortin 4 receptor	MC_51
122.	gi|189069046|dbj|BAG38486.1| melanocortin 4 receptor	MC_52
123.	gi|189069048|dbj|BAG38487.1| melanocortin 4	MC_53

	receptor	
124.	gi|189069050|dbj|BAG38488.1| melanocortin 4 receptor	MC_54
125.	gi|189069052|dbj|BAG38489.1| melanocortin 4 receptor	MC_55
126.	gi|189069054|dbj|BAG38490.1| melanocortin 4 receptor	MC_56
127.	gi|189069056|dbj|BAG38491.1| melanocortin 4 receptor	MC_57
128.	gi|189069058|dbj|BAG38492.1| melanocortin 4 receptor	MC_58
129.	gi|74096039|ref|NP_001027732.1| melanocortin 4 receptor	MC_59
130.	gi|28827154|gb|AAO24750.1| melanocortin 5 receptor	MC_60
131.	gi|29165382|gb|AAO65552.1| melanocortin 5 receptor	MC_61
132.	gi|29165384|gb|AAO65553.1| melanocortin 5 receptor	MC_62
133.	gi|74136149|ref|NP_001027937.1| melanocortin 5 receptor	MC_63
134.	gi|42559069|sp|Q9PUQ8.1|S1PR3_FUGRU RecName: Full=Sphingosine 1-phosphate receptor 3	EDGR_1
135.	gi|6409235|gb|AAF07896.1|AF164114_1 EDG-3	EDGR_2
136.	gi|1545938|emb|CAA64174.1| cannabinoid receptor type 1A	CNR_1
137.	gi|2494952|sp|Q98894.1|CNR1A_FUGRU RecName: Full=Cannabinoid receptor type 1A	CNR_2
138.	gi|1545940|emb|CAA64175.1| cannabinoid receptor type 1B	CNR_3

139.	gi|2494953|sp|Q98895.1|CNR1B_FUGRU RecName: Full=Cannabinoid receptor type 1B	CNR_4
140.	gi|32165506|gb|AAP72120.1| G protein-coupled receptor 119	MECAorph_1
141.	gi|74095967|ref|NP_001027835.1| G protein-coupled receptor 119	MECAorph_2
142.	tr|Q7T2L4|Q7T2L4_FUGRU G protein-coupled receptor 119 OS=Fugu rubripes GN=GPR119 PE=2 SV=1	MECAorph_3
143.	gi|116078050|dbj|BAF34888.1| neuropeptide FF receptor-1 NPFF2-1	NPFF_1
144.	gi|148839337|ref|NP_001092118.1| neuropeptide FF receptor-1 NPFF2-1	NPFF_2
145.	tr|Q05KN3|Q05KN3_FUGRU Neuropeptide FF receptor-1 NPFF2-1 OS=Fugu rubripes GN=NPFFR-1 PE=2 SV=1	NPFF_3
146.	gi|116078052|dbj|BAF34889.1| neuropeptide FF receptor-2 NPFF2-2	NPFF_4
147.	gi|148839282|ref|NP_001092119.1| neuropeptide FF receptor-2 NPFF2-2	NPFF_5
148.	gi|33304628|gb|AAQ02694.1| putative tachykinin receptor 1	TACR_1
149.	gi|156615963|gb|ABU87345.1| Npy2r protein	NPY_1
150.	gi|157412304|ref|NP_001098693.1| neuropeptide Y receptor 2	NPY_2
151.	gi|156615965|gb|ABU87346.1| Npy4r protein	NPY_3
152.	gi|156615967|gb|ABU87347.1| Npy7r protein	NPY_4
153.	gi|157412308|ref|NP_001098695.1| neuropeptide Y receptor Y7	NPY_5
154.	gi|156615969|gb|ABU87348.1| Npy8ar protein	NPY_6

155.	gi|156615971|gb|ABU87349.1| Npy8br protein	NPY_7
156.	gi|157265549|ref|NP_001098073.1| neuropeptide Y/peptide YY receptor	NPY_8
157.	gi|157265553|ref|NP_001098074.1| neuropeptide Y/peptide YY receptor Npy8br	NPY_9
158.	gi|157265555|ref|NP_001098075.1| neuropeptide Y/peptide YY receptor Npy4r	NPY_10
159.	gi|126148709|dbj|BAF47415.1| endothelin receptor type A	EdR_1
160.	gi|148839368|ref|NP_001092135.1| endothelin receptor type A	EdR_2
161.	gi|37540240|gb|AAK17004.1| vasotocin receptor V1-alpha	AVPR_1
162.	gi|37540242|gb|AAK18744.1| vasotocin receptor V1-beta	AVPR_2
163.	gi|116078048|dbj|BAF34887.1| RFamide-related peptide receptor	RF_1
164.	tr|Q05KN4|Q05KN4_FUGRU RFamide-related peptide receptor	RF_2
165.	gi|148839288|ref|NP_001092117.1| RFamide-related peptide receptor	RF_3
166.	gi|16945894|gb|AAL32173.1|AF329945_4 somatostatin receptor 2	SSTR_1
167.	gi|3122892|sp|O42179.1|SSRL_FUGRU RecName: Full=Somatostatin-like receptor	SSTR_2
168.	gi|2627438|gb|AAB86684.1| unknown	SSTR_3
169.	gi|28827164|gb|AAO24755.1| melanin-concentrating hormone receptor 1	MCH_1
170.	gi|28827166|gb|AAO24756.1| melanin-concentrating hormone receptor 2	MCH_2

171.	gi|65306815|gb|AAY41945.1| interleukin 8 receptor I transcript 1	CHE_1
172.	gi|118344648|ref|NP_001072110.1| interleukin 8 receptor I transcript 1	CHE_2
173.	gi|65306817|gb|AAY41946.1| interleukin 8 receptor I transcript 2	CHE_3
174.	gi|148233476|ref|NP_001091093.1| interleukin 8 receptor I transcript 2	CHE_4
175.	gi|65306819|gb|AAY41947.1| interleukin 8 receptor II	CHE_5
176.	gi|118344614|ref|NP_001072090.1| interleukin 8 receptor II	CHE_6
177.	gi|186928047|tpg|DAA06178.1| TPA_inf: luteinizing hormone receptor	LHCGR
178.	gi|32165504|gb|AAP72119.1| G protein-coupled receptor 100	LGR_1
179.	gi|74096007|ref|NP_001027859.1| G protein-coupled receptor 100	LGR_2
180.	tr|Q7T2L5|Q7T2L5_FUGRU G protein-coupled receptor 100	LGR_3
181.	gi|83752852|gb|ABC43425.1| odorant receptor	Odo_1
182.	gi|83752854|gb|ABC43426.1| odorant receptor	Odo_2
183.	gi|83752856|gb|ABC43427.1| odorant receptor	Odo_3
184.	gi|83752858|gb|ABC43428.1| odorant receptor	Odo_4
185.	gi|83752860|gb|ABC43429.1| odorant receptor	Odo_5
186.	gi|83752862|gb|ABC43430.1| odorant receptor	Odo_6
187.	gi|83752864|gb|ABC43431.1| odorant receptor	Odo_7
188.	gi|83752866|gb|ABC43432.1| odorant receptor	Odo_8
189.	gi|83752870|gb|ABC43434.1| odorant receptor	Odo_9

190.	gi|83752873|gb|ABC43435.1| odorant receptor	Odo_10
191.	gi|83752875|gb|ABC43436.1| odorant receptor	Odo_11
192.	gi|83752878|gb|ABC43437.1| odorant receptor	Odo_12
193.	gi|83752880|gb|ABC43438.1| odorant receptor	Odo_13
194.	gi|83752882|gb|ABC43439.1| odorant receptor	Odo_14
195.	gi|83752884|gb|ABC43440.1| odorant receptor	Odo_15
196.	gi|83752886|gb|ABC43441.1| odorant receptor	Odo_16
197.	gi|83752888|gb|ABC43442.1| odorant receptor	Odo_17
198.	gi|83752890|gb|ABC43443.1| odorant receptor	Odo_18
199.	gi|83752896|gb|ABC43445.1| odorant receptor	Odo_19
200.	gi|83752898|gb|ABC43446.1| odorant receptor	Odo_20
201.	gi|83752900|gb|ABC43447.1| odorant receptor	Odo_21
202.	gi|83752902|gb|ABC43448.1| odorant receptor	Odo_22
203.	gi|83752904|gb|ABC43449.1| odorant receptor	Odo_23
204.	gi|83752906|gb|ABC43450.1| odorant receptor	Odo_24
205.	gi|83752910|gb|ABC43452.1| odorant receptor	Odo_25
206.	gi|83752912|gb|ABC43453.1| odorant receptor	Odo_26
207.	gi|83752914|gb|ABC43454.1| odorant receptor	Odo_27
208.	gi|83752916|gb|ABC43455.1| odorant receptor	Odo_28
209.	gi|83752918|gb|ABC43456.1| odorant receptor	Odo_29
210.	gi|83752920|gb|ABC43457.1| odorant receptor	Odo_30
211.	gi|83752922|gb|ABC43458.1| odorant receptor	Odo_31
212.	gi|83752924|gb|ABC43459.1| odorant receptor	Odo_32
213.	gi|83752926|gb|ABC43460.1| odorant receptor	Odo_33

214.	gi|83752928|gb|ABC43461.1| odorant receptor	Odo_34
215.	gi|83752930|gb|ABC43462.1| odorant receptor	Odo_35
216.	gi|83752933|gb|ABC43463.1| odorant receptor	Odo_36
217.	gi|83752935|gb|ABC43464.1| odorant receptor	Odo_37
218.	gi|83752937|gb|ABC43465.1| odorant receptor	Odo_38
219.	gi|83752940|gb|ABC43466.1| odorant receptor	Odo_39
220.	gi|83752942|gb|ABC43467.1| odorant receptor	Odo_40
221.	gi|83752944|gb|ABC43468.1| odorant receptor	Odo_41
222.	gi|83752946|gb|ABC43469.1| odorant receptor	Odo_42
223.	gi|83752948|gb|ABC43470.1| odorant receptor	Odo_43
224.	gi|83752950|gb|ABC43471.1| odorant receptor	Odo_44
225.	gi|83752952|gb|ABC43472.1| odorant receptor [Takifugu rubripes]	Odo_45
226.	gi|83752954|gb|ABC43473.1| odorant receptor	Odo_46
227.	gi|83752956|gb|ABC43474.1| odorant receptor	Odo_47
228.	gi|7106161|dbj|BAA92165.1| olfactory receptor 1-1	Olf_1
229.	gi|7106163|dbj|BAA92166.1| olfactory receptor 1-2	Olf_2
230.	gi|7106165|dbj|BAA92167.1| olfactory receptor 1-3	Olf_3
231.	gi|7106167|dbj|BAA92168.1| olfactory receptor 1-4	Olf_4
232.	gi|7106169|dbj|BAA92169.1| olfactory receptor 1-5	Olf_5
233.	gi|7106171|dbj|BAA92170.1| olfactory receptor 2	Olf_6

234.	gi|5870765|gb|AAD54580.1|AF137214_1 rhodopsin	Undt_1
235.	gi|115305878|dbj|BAF32963.1| flg-Hepta	**A**|flg-H_1
236.	gi|148839278|ref|NP_001092114.1| flg-Hepta protein	**A**|flg-H_2
237.	lcl|scaffold_300.214923.216626 No definition line found	**FZD**|1
238.	lcl|scaffold_1549.10885.12627 No definition line found	**FZD**|2
239.	lcl|scaffold_536.20133.21884 No definition line found	**FZD**|3
240.	lcl|scaffold_2461.12412.14142 No definition line found	**FZD**|4
241.	lcl|scaffold_74.265266.266879 No definition line found	**FZD**|5
242.	lcl|scaffold_1909.3949.8009 No definition line found	**FZD**|6
243.	lcl|scaffold_43.402294.403766 No definition line found	**FZD**|7
244.	lcl|scaffold_6516.7.1682 No definition line found	**FZD**|8
245.	lcl|scaffold_849.34084.38418 No definition line found	**FZD**|9
246.	lcl|scaffold_3606.3197.10767 No definition line found	**FZD**|10
247.	gi|157101347|tpd|FAA00372.1| TPA: calcitonin receptor	CALCR_1
248.	gi|157412292|ref|NP_001098689.1| calcitonin receptor	CALCR_2
249.	gi|31043915|emb|CAC82924.2| corticotrophin releasing factor receptor	CRHR_1

250.	gi|23954212|emb|CAC82589.1| growth hormone releasing hormone-like peptide receptor	GHRHR_1
251.	gi|54400132|emb|CAD35690.1| pituitary adenylate-cyclase activating polypeptide receptor 1A	PACAP_1
252.	gi|54400134|emb|CAD38842.1| pituitary adenylate cyclase-activating polypeptide 1B	PACAP_2
253.	tr|Q5WML0|Q5WML0_FUGRU Pituitary adenylate cyclase-activating polypeptide 1B	PACAP_3
254.	tr|Q5WML1|Q5WML1_FUGRU Pituitary adenylate-cyclase activating polypeptide receptor 1A	PACAP_4
255.	gi|157412284|ref|NP_001098685.1| pituitary adenylate-cyclase activating polypeptide receptor 1A	PACAP_5
256.	gi|157412286|ref|NP_001098686.1| pituitary adenylate cyclase-activating polypeptide 1B	PACAP_6
257.	gi|83722082|emb|CAD67555.1| parathyroid hormone receptor 3	PTHR_1
258.	gi|83999576|emb|CAD68048.1| parathyroid hormone receptor	PTHR_2
259.	gi|83763923|emb|CAD79707.2| parathyroid hormone receptor 1	PTHR_3
260.	tr|Q2UZQ9|Q2UZQ9_FUGRU Parathyroid hormone receptor	PTHR_4
261.	tr|Q2UZQ8|Q2UZQ8_FUGRU Parathyroid hormone receptor 1	PTHR_5
262.	tr|Q2UZR0|Q2UZR0_FUGRU Parathyroid hormone receptor 3	PTHR_6
263.	gi|23954208|emb|CAC82587.1| vasoactive intestinal peptide receptor	VIPR_1
264.	gi|23954210|emb|CAC82588.1| vasoactive	VIPR_2

	intestinal peptide receptor 1 A	
265.	gi|48843195|emb|CAC83860.2| vasoactive intestinal peptide receptor	VIPR_3
266.	gi|48843196|emb|CAC83861.2| vasoactive intestinal peptide receptor	VIPR_4
267.	tr|Q802T6|Q802T6_FUGRU Vasoactive intestinal peptide receptor (Fragment)	VIPR_5
268.	tr|Q802T7|Q802T7_FUGRU Vasoactive intestinal peptide receptor (Fragment)	VIPR_6
269.	tr|Q8AXV3|Q8AXV3_FUGRU Vasoactive intestinal peptide receptor 1 A	VIPR_7
270.	tr|Q8AXV4|Q8AXV4_FUGRU Vasoactive intestinal peptide receptor	VIPR_8
271.	gi|34538247|gb|AAP04328.1| G-protein-coupled receptor GPR34 type 1	**OT7M**|1
272.	gi|32165510|gb|AAP72122.1| G protein-coupled receptor 142a	**OT7M**|2
273.	tr|Q7T2L2|Q7T2L2_FUGRU G protein-coupled receptor 142a (Fragment)	**OT7M**|3
274.	gi|32165512|gb|AAP72123.1| G protein-coupled receptor 142b	**OT7M**|4
275.	tr|Q7T2L1|Q7T2L1_FUGRU G protein-coupled receptor 142b (Fragment)	**OT7M**|5
276.	gi|32165608|gb|AAP72142.1| G protein-coupled receptor 135	**OT7M**|6
277.	gi|74095969|ref|NP_001027836.1| G protein-coupled receptor 135	**OT7M**|7
278.	tr|Q7T2L0|Q7T2L0_FUGRU G protein-coupled receptor 135	**OT7M**|8
279.	gi|118505345|gb|ABL01522.1| V1R pheromone receptor-like protein	**OT7M**|9

280.	gi|157412296|ref|NP_001098705.1| G-protein coupled receptor 39-1	**OT7M**|10
281.	gi|156147330|gb|ABU53899.1| G-protein coupled receptor 39-1a	**OT7M**|11
282.	gi|156147332|gb|ABU53900.1| G-protein coupled receptor 39-1b	**OT7M**|12
283.	gi|157412322|ref|NP_001098703.1| G protein-coupled receptor 155	**OT7M**|13
284.	gi|89114220|gb|ABD61705.1| progestin and adipoQ receptor family member VII	**OT7M**|14
285.	gi|109627672|ref|NP_001035912.1| progestin and adipoQ receptor family member VII	**OT7M**|15
286.	tr|Q19WU5|Q19WU5_FUGRU Progestin and adipoQ receptor family member VII	**OT7M**|16
287.	gi|94482840|gb|ABF22455.1| G protein-coupled receptor 155	**OT7M**|17
288.	gi|94482869|gb|ABF22483.1| G protein-coupled receptor 155	**OT7M**|18
289.	gi|3130165|dbj|BAA26128.1| metabotropic glutamate receptor 1 homologue	**G**|GRM_5
290.	gi|3130167|dbj|BAA26129.1| metabotropic glutamate receptor 2 homologue	**G**|GRM_6
291.	gi|3130169|dbj|BAA26130.1| metabotropic glutamate receptor 7 homologue	**G**|GRM_7
292.	gi|3130171|dbj|BAA26131.1| metabotropic glutamate receptor 8 homologue	**G**|GRM_8
293.	gi|3928164|emb|CAA09083.1| metabotropic glutamate receptor 7	**G**|GRM_9
294.	gi|85677367|dbj|BAE78486.1| taste receptor, type 1, member 1	**G**|TAS1_11
295.	gi|148234348|ref|NP_001091094.1| taste	**G**|TAS1_12

	receptor, type 1, member 1	
296.	gi|3130155|dbj|BAA26123.1| pheromone receptor	**G**|V2R_19
297.	gi|3130157|dbj|BAA26124.1| pheromone receptor	**G**|V2R_20
298.	gi|3130159|dbj|BAA26125.1| pheromone receptor	**G**|V2R_21
299.	gi|3130161|dbj|BAA26126.1| pheromone receptor	**G**|V2R_22
300.	gi|3130163|dbj|BAA26127.1| pheromone receptor	**G**|V2R_23
301.	gi|3130177|dbj|BAA26134.1| pheromone receptor	**G**|V2R_24
302.	gi|3130179|dbj|BAA26135.1| pheromone receptor	**G**|V2R_25
303.	gi|3130181|dbj|BAA26136.1| pheromone receptor	**G**|V2R_26
304.	gi|3130183|dbj|BAA26137.1| pheromone receptor	**G**|V2R_27
305.	gi|3130185|dbj|BAA26138.1| pheromone receptor	**G**|V2R_28
306.	gi|3130187|dbj|BAA26139.1| pheromone receptor	**G**|V2R_29
307.	gi|3130189|dbj|BAA26140.1| pheromone receptor	**G**|V2R_30
308.	gi|3130191|dbj|BAA26141.1| pheromone receptor	**G**|V2R_31
309.	gi|3130193|dbj|BAA26142.1| pheromone receptor	**G**|V2R_32
310.	gi|3130195|dbj|BAA26143.1| pheromone receptor	**G**|V2R_33

311.	gi|3130197|dbj|BAA26144.1| pheromone receptor	**G**|V2R_34
312.	gi|3130199|dbj|BAA26145.1| pheromone receptor	**G**|V2R_35
313.	gi|3130201|dbj|BAA26146.1| pheromone receptor	**G**|V2R_36
314.	gi|83752868|gb|ABC43433.1| odorant receptor	Odo_48
315.	gi|83752893|gb|ABC43444.1| odorant receptor	Odo_49
316.	gi|83752908|gb|ABC43451.1| odorant receptor	Odo_50
