# Supplementary material for: The G protein-coupled receptors in the pufferfish Takifugu rubripes
Source: BMC Bioinformatics. 2011 Feb 15;12(Suppl 1):S3. doi: 10.1186/1471-2105-12-S1-S3 (PMC3044285; doi:10.1186/1471-2105-12-S1-S3)
Supplement: Additional File 3 — Table enlisting the orthologs of Fugu GPCRs in the human, chicken and Tetraodon nigroviridis GPCR sub-sets. [file 1471-2105-12-S1-S3-S3.pdf]

The G Protein-Coupled Receptors in the pufferfish *Takifugu rubripes*

Anita Sarkar[#], Sonu Kumar[#] and Durai Sundar[*]

Department of Biochemical Engineering and Biotechnology, Indian Institute of Technology (IIT) Delhi, New Delhi, India

Supporting Information

Additional File 3. Table enlisting the orthologs of Fugu in human, chicken and *Tetraodon nigroviridis*.

S.No.	Acc. No. & Description	Query Length	Human		Chicken		*Tetraodon nigroviridis*	
			Identity	Gaps	Identity	Gaps	Identity	Gaps
I.	NCBI Sequences							
1.	AAB86684 (unknown, matched with somatostatin receptor 5 and unnamed protein product)	289	133/229(58%)	24/229(10%)	138/238 (57%)	35/238(14%)	185/227 (81%)	23/227 (10%)
2.	AAC96117 (putative neurotransmitter receptor, matched with trace amine associated receptor 1, 5 resp. and unnamed protein product)	328	101/290(34%)	21/290 (7%)	115/314 (36%)	18/314 (5%)	217/267 (81%)	0/267 (0%)
3.	AAC96118 (putative neurotransmitter receptor, matched with trace amine associated receptor 6, 5 and unnamed protein product)	328	115/331(34%)	15/331 (4%)	115/325 (35%)	14/325 (4%)	218/261 (83%)	0/261 (0%)
4.	AAD54580 (rhodopsin, matched with rhodopsin and unnamed protein product)	270	220/268(82%)	0/268 (0%)	221/270 (81%)	0/270 (0%)	254/270 (94%)	0/270 (0%)
5.	AAF07896 (EDG-3 , matched with sphingosine-1-	384	214/305(70%)	3/305 (0%)	220/312(70%),	4/312 (1%)	295/329 (89%)	0/329 (0%)

	phosphate receptor 3, endothelial differentiation, sphingolipid G-protein-coupled receptor, 3 and unnamed protein product)							
6.	AAF44621 (rod opsin, matched with rhodopsin and unnamed protein product)	353	262/327(80%)	0/327 (0%)	265/327 (81%)	0/327 (0%)	328/346 (94%)	0/346 (0%)
7.	AAF44622 (rod-like opsin, matched with rhodopsin and unnamed protein product)	353	262/332(78%)	0/332 (0%)	265/332 (79%)	0/332 (0%)	340/353 (96%)	0/353 (0%)
8.	AAF44648 (green opsin, matched with rhodopsin, rhodopsin (opsin 2, rod pigment) (retinitis pigmentosa 4, autosomal dominant) and green-sensitive pigment)	352	229/345(66%)	2/345 (0%)	237/342(69%)	4/342 (1%)	330/352 (93%)	1/352 (0%)
9.	AAK17004(vasotocin receptor V1-alpha, matched with arginine vasopressin receptor 1A and unnamed protein product)	447	211/349(60%)	8/349 (2%)	227/337(67%)	15/337 (4%)	240/345 (69%)	12/345 (3%)
10.	AAK18744 (vasotocin receptor V1-beta, matched with arginine vasopressin receptor 1A and unnamed protein product)	411	233/398(58%)	12/398 (3%)	247/374 (66%)	7/374 (1%)	344/382 (90%)	2/382 (0%)
11.	AAL32173 (somatostatin receptor 2 , matched with somatostatin receptor 2 and unnamed protein product)	370	256/369(69%)	6/369 (1%)	247/367(67%)	9/367 (2%)	178/318 (55%)	24/318 (7%)
12.	AAL83430 (TMT opsin, matched with opsin 3, similar to multiple tissue opsin and	310	102/253(40%)	9/253 (3%)	177/280(63%)	1/280 (0%)	206/236 (87%)	2/236 (0%)

	unnamed protein product)							
13.	AAM90677 (multiple tissue opsin, matched with opsin 3, similar to multiple tissue opsin and unnamed protein product)	402	103/254(40%)	9/254 (3%)	178/281(63%)	1/281 (0%)	206/236 (87%)	2/236 (0%)
14.	AAO24748 (melanocortin 2 receptor, matched with adrenocorticotropic hormone receptor, ACTH receptor, melanocortin 2 receptor adrenocorticotropic hormone and Melanocortin 2 receptor)	301	144/295(48%)	12/295 (4%)	147/298 (49%)	8/298 (2%)	266/299 (88%)	0/299 (0%)
15.	AAO24749 (melanocortin 1 receptor, matched with melanocortin 1 receptor and Melanocortin 1 receptor)	300	170/295(57%)	3/295 (1%)	188/297 (63%)	2/297 (0%)	272/300 (90%)	1/300 (0%)
16.	AAO24750 (melanocortin 5 receptor , matched with melanocortin 5 receptor and Melanocortin 5 receptor)	340	227/294(77%)	0/294 (0%)	246/323 (76%)	7/323 (2%)	304/340 (89%)	0/340 (0%)
17.	AAO24751 (melanocortin 4 receptor, matched with melanocortin 4 receptor and Melanocortin 4 receptor)	322	219/302(72%)	7/302 (2%)	222/323 (68%)	7/323 (2%)	303/323 (93%)	1/323 (0%)
18.	AAO24755 (melanin-concentrating hormone receptor 1, matched with melanin-concentrating hormone receptor 1 and unnamed protein product)	331	187/304(61%)	3/304 (0%)	212/335 (63%)	7/335 (2%)	259/287 (90%)	0/287 (0%)
19.	AAO24756 (melanin-concentrating hormone receptor 2, matched with melanin-concentrating hormone receptor 1 and	360	118/316(37%)	25/316 (7%)	103/282 (36%)	11/282 (3%)	320/339 (94%)	0/339 (0%)

	unnamed protein product)							
20.	AAO65548 (melanocortin 1 receptor, matched with melanocortin 1 receptor and Melanocortin 1 receptor)	300	170/295(57%)	3/295 (1%)	188/297 (63%)	2/297 (0%)	272/300 (90%)	1/300 (0%)
21.	AAO65549 (melanocortin 2 receptor, matched with adrenocorticotropic hormone receptor, ACTH receptor, melanocortin 2-receptor and Melanocortin 2 receptor)	301	144/295(48%)	12/295 (4%)	147/298 (49%)	8/298 (2%)	266/299 (88%)	0/299 (0%)
22.	AAO65550 (melanocortin 2 receptor, matched with adrenocorticotropic hormone receptor, ACTH receptor and Melanocortin 2 receptor)	301	144/295(48%)	12/295 (4%)	147/298 (49%)	8/298 (2%)	266/299 (88%)	0/299 (0%)
23.	AAO65551 (melanocortin 4 receptor, matched with melanocortin4 receptor and Melanocortin 4 receptor)	322	219/302(72%)	7/302 (2%)	222/323 (68%)	7/323 (2%)	303/323 (93%)	1/323 (0%)
24.	AAO65552 (melanocortin 5 receptor, matched melanocortin 5 receptor and Melanocortin 5 receptor)	340	227/294(77%)	0/294 (0%)	246/323 (76%)	7/323 (2%)	304/340 (89%)	0/340 (0%)
25.	AAO65553 (melanocortin 5 receptor, matched with melanocortin 5 receptor and Melanocortin 5 receptor)	340	227/294(77%)	0/294 (0%)	246/323 (76%)	7/323 (2%)	304/340 (89%)	0/340 (0%)
26.	AAP04328 (G-protein-coupled receptor GPR34 type 1, matched with G protein-coupled receptor 34 and G-protein-coupled receptor GPR34 type 1)	378	143/350(40%)	15/350 (4%)	143/350 (40%)	15/350 (4%)	258/365 (70%)	4/365 (1%)

27.	AAP72119 (G protein-coupled receptor 100, matched with relaxin/insulin-like family peptide receptor 3 and unnamed protein product)	408	231/386(59%)	35/386 (9%)	244/376 (64%)	22/376 (5%)	314/349 (89%)	0/349 (0%)
28.	AAP72120 (G protein-coupled receptor 119, matched with G protein-coupled receptor 119 and unnamed protein product)	393	131/315(41%)	30/315 (9%)	115/317 (36%)	39/317(12%)	249/337 (73%)	27/337 (8%)
29.	AAP72121 (G protein-coupled receptor 136, matched with G protein-coupled receptor 136 in human & opsin 5 in chicken and unnamed protein product)	259	182/313(58%)	56/313(17%)	173/316 (54%),	59/316(18%)	175/195 (89%)	7/195 (3%)
30.	AAP72122 (G protein-coupled receptor 142a, matched with G protein-coupled receptor 142, kinesin family member 19 and unnamed protein product)	371	172/296(58%)	11/296 (3%)	180/308 (58%)	19/308 (6%)	293/315 (93%)	6/315 (1%)
31.	AAP72123 (G protein-coupled receptor 142b, matched with G protein-coupled receptor 139 and unnamed protein product)	383	245/377(64%)	25/377 (6%)	243/380 (63%)	33/380 (8%)	311/328 (94%)	1/328 (0%)
32.	AAP72142 (G protein-coupled receptor 135, matched with G protein-coupled receptor 135 and unnamed protein product)	444	200/358(55%)	13/358 (3%)	177/299 (59%)	10/299 (3%)	238/257 (92%)	0/257 (0%)
33.	AAQ02694 (putative tachykinin receptor 1, matched with tachykinin receptor 1 and unnamed protein product)	393	251/385(65%)	15/385 (3%)	252/396 (63%)	11/396 (2%)	329/362 (90%)	0/362 (0%)

34.	AAQ02695 (putative beta-2 adrenergic receptor, matched with adrenergic, beta-2-, receptor, surface and unnamed protein product)	417	224/406(55%)	14/406 (3%)	225/378 (59%)	20/378 (5%)	340/357 (95%)	0/357 (0%)
35.	AAT38456 (red-sensitive pigment, matched with opsin 1 (cone pigments), long-wave-sensitive and red-sensitive pigment)	357	277/344(80%)	3/344 (0%)	276/326 (84%)	0/326 (0%)	335/357 (93%)	0/357 (0%)
36.	AAT38459 (blue-sensitive pigment, matched with opsin 1 (cone pigments), short-wave-sensitive, blue sensitive cone opsin and unnamed protein product)	351	170/343(49%)	6/343 (1%)	215/320 (67%)	0/320 (0%)	319/352 (90%)	1/352 (0%)
37.	AAU09270 (M2 muscarinic acetylcholine receptor, matched with cholinergic receptor, muscarinic 2 and unnamed protein product)	500	327/481(67%)	35/481 (7%)	341/502 (67%)	44/502 (8%)	377/498 (75%)	57/498 (11%)
38.	AAY41945 (interleukin 8 receptor I transcript 1, matched with interleukin 8 receptor, alpha and interleukin 8 receptor, beta and unnamed protein product)	352	111/263(42%)	3/263 (1%)	111/263 (42%)	3/263 (1%)	262/318 (82%)	4/318 (1%)
39.	AAY41946 (interleukin 8 receptor I transcript 2, matched with interleukin 8 receptor, alpha and unnamed protein product)	352	111/263(42%)	3/263 (1%)	111/263 (42%)	3/263 (1%)	262/318 (82%)	4/318 (1%)
40.	AAY41947 (interleukin 8 receptor II, matched with interleukin 8 receptor, alpha and interleukin 8 receptor,	359	141/336(41%)	23/336 (6%)	102/236 (43%)	2/236 (0%)	158/308 (51%)	3/308 (0%)

	beta and unnamed protein product)							
41.	ABC43425 (odorant receptor, matched with olfactory receptor, family 52, subfamily N, member 4 and olfactory receptor, family 52, subfamily R, member 1 and and unnamed protein product)	314	98/306 (32%)	6/306 (1%)	100/315 (31%)	11/315 (3%)	177/219 (80%)	0/219 (0%)
42.	ABC43426 (odorant receptor, matched with olfactory receptor, family 51, subfamily D, member 1 and odorant receptor)	256	63/231 (27%)	33/231(14%)	No significant similarity found.		186/252 (73%)	0/252 (0%)
43.	ABC43427 (odorant receptor, matched with olfactory receptor, family 5, subfamily F, member 1 and similar to olfactory receptor 5 and odorant receptor)	314	98/312 (31%)	14/312 (4%)	89/268 (33%)	9/268 (3%)	160/305 (52%)	1/305 (0%)
44.	ABC43428 (odorant receptor, matched with olfactory receptor, family 10, subfamily J, member 4 (gene/pseudogene) and olfactory receptor OR16 and odorant receptor)	308	90/288 (31%)	12/288 (4%)	87/279 (31%)	11/279 (3%)	251/308 (81%)	0/308 (0%)
45.	ABC43429 (odorant receptor, matched with hCG1645405 and similar to olfactory receptor, family 52, subfamily R, member 1 and odorant receptor)	320	76/258 (29%)	2/258 (0%)	81/264 (30%)	9/264 (3%)	244/319 (76%)	1/319 (0%)
46.	ABC43430 (odorant receptor, matched with olfactory receptor, family 4, subfamily N, member 2 and olfactory	310	83/249 (33%)	5/249 (2%)	86/256 (33%)	7/256 (2%)	183/304 (60%)	0/304 (0%)

	receptor, family 12, subfamily D, member 2 and odorant receptor)							
47.	ABC43431 (odorant receptor, matched with olfactory receptor, family 6, subfamily K, member 3 and similar to olfactory receptor Olr649 and odorant receptor)	308	97/314 (30%)	17/314 (5%)	95/307 (30%)	8/307 (2%)	248/308 (80%)	1/308 (0%)
48.	ABC43432 (odorant receptor, matched with olfactory receptor, family 2, subfamily T, member 27 and similar to olfactory receptor MOR260-5 and unnamed protein product)	312	91/309 (29%)	4/309 (1%)	96/280 (34%)	9/280 (3%)	165/206 (80%)	0/206 (0%)
49.	ABC43434 (odorant receptor, matched with olfactory receptor, family 7, subfamily C, member 2 and olfactory receptor, family 52, subfamily R, member 1 and unnamed protein product)	312	88/284 (30%)	10/284 (3%)	91/279 (32%)	4/279 (1%)	205/262 (78%)	0/262 (0%)
50.	ABC43435 (odorant receptor, matched with olfactory receptor, family 52, subfamily E, member 8 and similar to olfactory receptor Olr202 and odorant receptor)	321	107/298(35%)	3/298 (1%)	105/299 (35%)	2/299 (0%)	267/319 (83%)	0/319 (0%)
51.	ABC43436 (odorant receptor, matched with olfactory receptor, family 52, subfamily E, member 8 and similar to olfactory receptor Olr202 and odorant receptor)	319	103/296(34%)	4/296 (1%)	88/269 (32%)	0/269 (0%)	184/316 (58%)	2/316 (0%)
52.	ABC43437 (odorant receptor and odorant receptor)	309	No significant similarity found.		No significant similarity found.		101/274 (36%)	4/274 (1%)

53.	ABC43438 (odorant receptor, matched with olfactory receptor, family 7, subfamily C, member 2 and olfactory receptor OR35 and odorant receptor)	313	99/307 (32%)	10/307 (3%)	96/309 (31%)	15/309 (4%)	228/308 (74%)	0/308 (0%)
54.	ABC43439 (odorant receptor, matched with olfactory receptor, family 11, subfamily H, member 6 and similar to olfactory receptor MOR181-2 and odorant receptor)	312	97/298 (32%)	11/298 (3%)	104/321 (32%)	19/321 (5%)	138/295 (46%)	0/295 (0%)
55.	ABC43440 (odorant receptor, matched with olfactory receptor, family 52, subfamily H, member 1 and similar to olfactory receptor, family 52, subfamily R, member 1 and unnamed protein product)	304	96/311 (30%)	17/311 (5%)	86/251 (34%)	5/251 (1%)	146/304 (48%)	0/304 (0%)
56.	ABC43441 (odorant receptor, matched with olfactory receptor, family 51, subfamily E, member 1 and olfactory receptor, family 52, subfamily B, member 2 and odorant receptor)	307	89/300 (29%)	13/300 (4%)	87/284 (30%)	28/284 (9%)	167/300 (55%)	2/300 (0%)
57.	ABC43442 (odorant receptor, matched with olfactory receptor, family 52, subfamily E, member 4 and similar to olfactory receptor 1320 and olfactory receptor, family 1, subfamily D, member 2 and odorant receptor)	311	87/288 (30%)	5/288 (1%)	90/303 (29%)	5/303 (1%)	252/309 (81%)	1/309 (0%)
58.	ABC43443 (odorant receptor, matched with olfactory	306	87/242 (35%)	4/242 (1%)	81/249 (32%)	20/249 (8%)	229/306 (74%)	0/306 (0%)

	receptor, family 1, subfamily D, member 2 and similar to olfactory receptor MOR254-1 and odorant receptor)							
59.	ABC43445 (odorant receptor, matched with olfactory receptor, family 4, subfamily H, member 12 pseudogene andsimilar to olfactory receptor MOR181-2 and odorant receptor)	308	99/302 (32%)	24/302 (7%)	95/310 (30%)	29/310 (9%)	235/307 (76%)	0/307 (0%)
60.	ABC43446 (odorant receptor, matched with olfactory receptor, family 7, subfamily C, member 1 and similar to olfactory receptor MOR181-2 and odorant receptor)	306	97/309 (31%)	9/309 (2%)	107/312 (34%)	15/312 (4%)	232/305 (76%)	0/305 (0%)
61.	ABC43447 (odorant receptor, matched with olfactory receptor, family 7, subfamily C, member 1 and similar to olfactory receptor MOR254-1 and odorant recptor)	309	89/253 (35%)	8/253 (3%)	85/250 (34%)	18/250 (7%)	221/308 (71%)	2/308 (0%)
62.	ABC43448 (odorant receptor, matched with olfactory receptor, family 1, subfamily C, member 1 and similar to chick olfactory receptor 7 and odorant receptors)	335	89/261 (34%)	5/261 (1%)	102/310 (32%)	2/310 (0%)	294/335 (87%)	0/335 (0%)
63.	ABC43449 (odorant receptor, matched with olfactory receptor, family 7, subfamily C, member 1 and similar to olfactory receptor MOR181-2 and odorant receptor)	316	84/243 (34%)	4/243 (1%)	90/267 (33%)	12/267 (4%)	221/306 (72%)	0/306 (0%)
64.	ABC43450 (odorant receptor)	257	No significant similarity found.		No significant similarity found.		160/251 (63%)	1/251 (0%)

65.	ABC43452 (odorant receptor and odorant receptor)	299	No significant similarity found.		No significant similarity found.		217/262 (82%)	0/262 (0%)
66.	ABC43453 (odorant receptor, matched with olfactory receptor, family 11, subfamily A, member 1 and similar to olfactory receptor Olr649 and odorant receptor)	303	101/306(33%)	16/306 (5%)	93/293 (31%)	12/293 (4%)	152/302 (50%)	0/302 (0%)
67.	ABC43454 (odorant receptor, matched with olfactory receptor, family 4, subfamily S, member 1 and similar to olfactory receptor Olr649 and odorant receptor)	316	90/318 (28%)	25/318 (7%)	83/291 (28%)	15/291 (5%)	181/306 (59%)	7/306 (2%)
68.	ABC43455 (odorant receptor, matched with olfactory receptor, family 1, subfamily M, member 1 and olfactory receptor, family 12, subfamily D, member 2 and odorant receptor)	237	71/204 (34%)	2/204 (0%)	77/237 (32%)	7/237 (2%)	196/236 (83%)	0/236 (0%)
69.	ABC43456 (odorant receptor, matched with olfactory receptor, family 52, subfamily K, member 1 and similar to olfactory receptor MOR254-1 and odorant receptor)	323	96/306 (31%)	6/306 (1%)	92/301 (30%)	3/301 (0%)	268/322 (83%)	0/322 (0%)
70.	ABC43457 (odorant receptor, matched with olfactory receptor, family 52, subfamily J, member 3 and olfactory receptor, family 52, subfamily R, member 1 and unnamed protein product)	317	85/282 (30%)	12/282 (4%)	91/305 (29%)	11/305 (3%)	121/217 (55%)	0/217 (0%)

71.	ABC43458 (odorant receptor, matched with seven transmembrane helix receptor and olfactory receptor, family 52, subfamily R, member 1 and unnamed protein product)	314	86/262 (32%)	4/262 (1%)	84/254 (33%)	9/254 (3%)	189/219 (86%)	0/219 (0%)
72.	ABC43459 (odorant receptor, matched with olfactory receptor, family 52, subfamily E, member 4 and olfactory receptor, family 52, subfamily R, member 1 and odorant receptor)	256	86/256 (33%)	6/256 (2%)	86/258 (33%)	7/258 (2%)	203/258 (78%)	3/258 (1%)
73.	ABC43460 (odorant receptor, matched with olfactory receptor, family 52, subfamily D, member 1 and similar to olfactory receptor Olr202 and odorant receptor)	324	124/314(39%)	2/314 (0%)	128/308 (41%)	1/308 (0%)	285/324 (87%)	0/324 (0%)
74.	ABC43461 (odorant receptor, matched with olfactory receptor, family 11, subfamily A, member 1 and similar to olfactory receptor MOR104-3 and odorant receptor)	318	97/301 (32%)	18/301 (5%)	94/296 (31%)	10/296 (3%)	230/305 (75%)	0/305 (0%)
75.	ABC43462 (odorant receptor, matched with olfactory receptor, family 2, subfamily A, member 14 and similar to olfactory receptor, family 52, subfamily R, member 1 and unnamed protein product)	305	88/255 (34%)	9/255 (3%)	86/255 (33%)	11/255 (4%)	236/305 (77%)	0/305 (0%)
76.	ABC43463 (odorant receptor and odorant receptor)	317	No significant similarity found		No significant similarity found		249/317 (78%)	0/317 (0%)

77.	ABC43464 (odorant receptor and odorant receptor)	317	No significant similarity found		No significant similarity found		243/317 (76%)	0/317 (0%)
78.	ABC43465 (odorant receptor, matched with olfactory receptor, family 1, subfamily F, member 1 and olfactory receptor, family 12, subfamily D, member 2 and odorant receptor)	323	87/289 (30%)	3/289 (1%)	90/291 (30%)	7/291 (2%)	269/322 (83%)	0/322 (0%)
79.	ABC43466 (odorant receptor, matched with olfactory receptor, family 7, subfamily C, member 1 and similar to olfactory receptor MOR181-2 and odorant receptor)	306	85/246 (34%)	14/246 (5%)	81/241 (33%)	4/241 (1%)	219/305 (71%)	0/305 (0%)
80.	ABC43467 (odorant receptor, matched with galanin receptor 2 and odorant receptor)	312	No significant similarity found		58/245 (23%)	39/245 (15%)	149/277 (53%)	0/277 (0%)
81.	ABC43468 (odorant receptor, matched with olfactory receptor, family 7, subfamily C, member 1 and similar to olfactory receptor MOR181-2 and odorant receptor)	313	63/146 (43%)	0/146 (0%)	99/308 (32%)	7/308 (2%)	228/306 (74%)	0/306 (0%)
82.	ABC43469 (odorant receptor, matched with olfactory receptor, family 7, subfamily C, member 2 and olfactory receptor OR35 and odorant receptor)	313	99/307 (32%)	10/307 (3%)	96/309 (31%)	15/309 (4%)	231/308 (75%)	0/308 (0%)
83.	ABC43470 (odorant receptor and odorant receptor)	314	No significant similarity found		No significant similarity found		191/243 (78%)	0/243 (0%)

84.	ABC43471 (odorant receptor, matched with olfactory receptor, family 1, subfamily L, member 6 and olfactory receptor, family 52, subfamily R, member 1 and odorant receptor)	308	92/302 (30%)	22/302 (7%)	87/259 (33%)	2/259 (0%)	216/308 (70%)	2/308 (0%)
85.	ABC43472 (odorant receptor and odorant receptor)	309	No significant similarity found		No significant similarity found		193/279 (69%)	0/279 (0%)
86.	ABC43473 (odorant receptor, matched with hCG1645405 and olfactory receptor, family 52, subfamily R, member 1 and odorant receptor)	334	93/307 (30%)	4/307 (1%)	96/304 (31%)	11/304 (3%)	179/219 (81%)	0/219 (0%)
87.	ABC43474 (odorant receptor, matched with olfactory receptor, family 11, subfamily A, member 1 and olfactory receptor, family 10, subfamily A, member 4 and odorant receptor)	308	96/303 (31%)	18/303 (5%)	92/299 (30%)	17/299 (5%)	233/307 (75%)	0/307 (0%)
88.	ABD61705 (progestin and adipoQ receptor family member VII, matched with progestin and adipoQ receptor family member VIII and progestin and adipoQ receptor family member VII and unnamed protein product)	352	175/354(49%)	2/354 (0%)	184/324 (56%)	6/324 (1%)	342/350 (97%)	0/350 (0%)
89.	ABL01522 (V1R pheromone receptor-like protein, matched with vomeronasal 1 receptor 1 and V1R pheromone receptor-like)	327	77/299 (25%)	5/299 (1%)	No significant similarity found		271/321 (84%)	2/321 (0%)

90.	ABU53899 (G-protein coupled receptor 39-1a, matched with G protein-coupled receptor 39 and unnamed protein product)	417	197/376(52%)	24/376 (6%)	195/368 (52%)	22/368 (5%)	242/244 (99%)	0/244 (0%)
91.	ABU53900 (G-protein coupled receptor 39-1b, matched with G protein-coupled receptor 39 and G protein-coupled receptor 39-1b and unnamed protein product)	322	139/280(49%)	23/280 (8%)	138/284 (48%)	22/284 (7%)	301/301 (100%)	0/301 (0%)
92.	ABU87345 (Npy2r protein, matched with neuropeptide Y receptor Y2 and unnamed protein product)	396	201/306(65%)	5/306 (1%)	203/316 (64%)	7/316 (2%)	309/339 (91%)	0/339 (0%)
93.	ABU87346 (Npy4r protein, matched with neuropeptide Y receptor Y1 and neuropeptide Y receptor 4 and unnamed protein product)	411	172/368(46%)	11/368 (2%)	190/374 (50%)	14/374 (3%)	263/291 (90%)	4/291 (1%)
94.	ABU87347 (Npy7r protein, matched with neuropeptide Y receptor Y2 and neuropeptide Y7 receptor and unnamed protein product)	369	202/362(55%)	7/362 (1%)	240/339 (70%)	5/339 (1%)	172/301 (57%)	7/301 (2%)
95.	ABU87348 (Npy8ar protein, matched with neuropeptide Y receptor Y1 and pancreatic polypeptide receptor 1 and unnamed protein product)	406	169/354(47%)	28/354 (7%)	178/363 (49%)	26/363 (7%)	278/304 (91%)	21/304 (6%)
96.	ABU87349 (Npy8br protein, matched with neuropeptide Y receptor Y1 and NPY receptor Y6 and unnamed protein product)	364	174/337(51%)	3/337 (0%)	184/346 (53%)	3/346 (0%)	305/345 (88%)	0/345 (0%)
97.	BAA92165 (olfactory receptor 1-1, matched with olfactory	323	88/289 (30%)	3/289 (1%)	96/292 (32%)	7/292 (2%)	268/322	0/322

	receptor, family 1, subfamily F, member 1 and olfactory receptor, family 12, subfamily D, member 2 and unnamed protein product)						(83%)	(0%)
98.	BAA92166 (olfactory receptor 1-2, matched with olfactory receptor, family 52, subfamily K, member 1 and similar to olfactory receptor MOR254-1 and odorant receptor)	323	97/314 (30%)	19/314 (6%)	92/301 (30%)	3/301 (0%)	268/322 (83%)	0/322 (0%)
99.	BAA92167 (olfactory receptor 1-3, matched with olfactory receptor, family 1, subfamily F, member 1 and olfactory receptor, family 12, subfamily D, member 2 and odorant receptor)	323	88/289 (30%)	3/289 (1%)	90/291 (30%)	7/291 (2%)	269/322 (83%)	0/322 (0%)
100.	BAA92168 (olfactory receptor 1-4, matched with olfactory receptor, family 1, subfamily F, member 1 and olfactory receptor, family 12, subfamily D, member 2 and odorant receptor)	323	90/289 (31%)	3/289 (1%)	93/292 (31%)	7/292 (2%)	264/322 (81%)	0/322 (0%)
101.	BAA92169 (olfactory receptor 1-5, matched with olfactory receptor, family 51, subfamily D, member 1 and olfactory receptor, family 12, subfamily D, member 2 and odorant receptor)	323	106/329(32%)	38/329(11%)	89/291 (30%)	7/291 (2%)	271/322 (84%)	0/322 (0%)
102.	BAA92170 (olfactory receptor 2 , matched with olfactory receptor, family 52, subfamily E, member 4 and olfactory receptor, family 52, subfamily R, member 1 and odorant	319	89/261 (34%)	6/261 (2%)	89/263 (33%)	7/263 (2%)	225/292 (77%)	3/292 (1%)

	receptor)							
103.	BAB71730 (melanocortin receptor-4, matched with melanocortin 4 receptor and Melanocortin 4 receptor)	322	219/302(72%)	7/302 (2%)	222/323 (68%)	7/323 (2%)	303/323 (93%)	1/323 (0%)
104.	BAE78487 (taste receptor, type 1, member 2a, matched with taste receptor, type 1, member 1 and unnamed protein product)	824	291/833(34%)	26/833 (3%)	222/667 (33%	30/667 (4%)	671/838 (80%)	23/838 (2%)
105.	BAE78488 (taste receptor, type 1, member 2b , matched with taste receptor, type 1, member 1 and unnamed protein product)	816	304/821(37%)	40/821 (4%)	242/668 (36%)	35/668 (5%)	541/738 (73%)	1/738 (0%)
106.	BAE78489 (taste receptor, type 1, member 3 , matched with taste receptor, type 1, member 3 and unnamed protein product)	853	306/808(37%)	17/808 (2%)	309/806 (38%)	16/806 (1%)	678/829 (81%)	0/829 (0%)
107.	BAF32963 (flg-Hepta , matched with G protein-coupled receptor 116 and unnamed protein product)	1678	276/801(34%)	89/801(11%)	257/708 (36%)	73/708(10%)	150/312 (48%)	32/312 (10%)
108.	BAF34887 (RFamide-related peptide receptor, matched with neuropeptide FF receptor 2 and neuropeptide FF receptor 1 and unnamed protein product)	474	196/406(48%)	31/406 (7%)	204/364 (56%)	15/364 (4%)	294/339 (86%)	0/339 (0%)
109.	BAF34888 (neuropeptide FF receptor-1 NPFF2-1 , matched with neuropeptide FF receptor 2 and unnamed protein product)	426	262/403(65%)	1/403 (0%)	298/416 (71%)	2/416 (0%)	324/389 (83%)	43/389 (11%)

110.	BAF34889 (neuropeptide FF receptor-2 NPFF2-2 , matched with neuropeptide FF receptor 2 and unnamed protein product)	453	245/422(58%)	41/422 (9%)	259/416 (62%)	29/416 (6%)	316/398 (79%)	42/398 (10%)
111.	BAF47415 (endothelin receptor type A , matched with endothelin receptor type A and unnamed protein product)	468	238/380(62%)	26/380 (6%)	246/403 (61%)	32/403 (7%)	289/384 (75%)	32/384 (8%)
112.	BAG38415 (melanocortin 1 receptor, matched with melanocortin 1 receptor and melanocortin 1 receptor)	300	170/295(57%)	3/295 (1%)	188/297 (63%)	2/297 (0%)	272/300 (90%)	1/300 (0%)
113.	BAG38416 (melanocortin 1 receptor, matched with melanocortin 1 receptor and melanocortin receptor 1)	300	170/295(57%)	3/295 (1%)	188/297 (63%)	2/297 (0%)	272/300 (90%)	1/300 (0%)
114.	BAG38417 (melanocortin 1 receptor, matched with melanocortin 1 receptor and melanocortin 1 receptor (alpha melanocyte stimulating hormone receptor) and melanocortin 1 receptor)	300	170/295(57%)	3/295 (1%)	188/297 (63%)	2/297 (0%)	272/300 (90%)	1/300 (0%)
115.	BAG38418 (melanocortin 1 receptor, matched with melanocortin 1 receptor and melanocortin receptor 1)	300	170/295(57%)	3/295 (1%)	188/297 (63%)	2/297 (0%)	272/300 (90%)	1/300 (0%)
116.	BAG38419 (melanocortin 1 receptor, matched with melanocortin 1 receptor and melanocortin receptor 1)	300	170/295(57%)	3/295 (1%)	188/297 (63%)	2/297 (0%)	272/300 (90%)	1/300 (0%)
117.	BAG38420 (melanocortin 1 receptor, matched with melanocortin 1 receptor and	300	170/295(57%)	3/295 (1%)	188/297 (63%)	2/297 (0%)	272/300 (90%)	1/300 (0%)

	melanocortin 1 receptor (alpha melanocyte stimulating hormone receptor) and melanocortin receptor 1)							
118.	BAG38421 (melanocortin 1 receptor, matched with melanocortin 1 receptor and melanocortin 1 receptor (alpha melanocyte stimulating hormone receptor) and melanocortin receptor 1)	300	170/295(57%)	3/295 (1%)	188/297 (63%)	2/297 (0%)	272/300 (90%)	1/300 (0%)
119.	BAG38422 (melanocortin 1 receptor, matched with melanocortin 1 receptor and melanocortin 1 receptor (alpha melanocyte stimulating hormone receptor) and melanocortin receptor 1)	300	170/295(57%)	3/295 (1%)	188/297 (63%)	2/297 (0%)	272/300 (90%)	1/300 (0%)
120.	BAG38423 (melanocortin 1 receptor, matched with melanocortin 1 receptor and melanocortin receptor 1)	300	170/295(57%)	3/295 (1%)	188/297 (63%)	2/297 (0%)	272/300 (90%)	1/300 (0%)
121.	BAG38424 (melanocortin 1 receptor, matched with melanocortin 1 receptor and melanocortin 1 receptor (alpha melanocyte stimulating hormone receptor) and melanocortin receptor 1)	300	170/295(57%)	3/295 (1%)	188/297 (63%)	2/297 (0%)	272/300 (90%)	1/300 (0%)
122.	BAG38425 (melanocortin 1 receptor, matched with melanocortin 1 receptor and melanocortin 1 receptor (alpha melanocyte	300	170/295(57%)	3/295 (1%)	188/297 (63%)	2/297 (0%)	272/300 (90%)	1/300 (0%)

	stimulating hormone receptor) and melanocortin receptor 1)							
123.	BAG38426 (melanocortin 1 receptor, matched with melanocortin 1 receptor and melanocortin 1 receptor (alpha melanocyte stimulating hormone receptor) and melanocortin receptor 1)	300	170/295(57%)	3/295 (1%)	188/297 (63%)	2/297 (0%)	272/300 (90%)	1/300 (0%)
124.	BAG38427 (melanocortin 1 receptor, matched with melanocortin 1 receptor and melanocortin 1 receptor (alpha melanocyte stimulating hormone receptor) and melanocortin receptor 1)	300	170/295(57%)	3/295 (1%)	188/297 (63%)	2/297 (0%)	272/300 (90%)	1/300 (0%)
125.	BAG38428 (melanocortin 1 receptor, matched with melanocortin 1 receptor and melanocortin 1 receptor (alpha melanocyte stimulating hormone receptor) and melanocortin receptor 1)	300	170/295(57%)	3/295 (1%)	188/297 (63%)	2/297 (0%)	272/300 (90%)	1/300 (0%)
126.	BAG38429 (melanocortin 1 receptor, matched with melanocortin 1 receptor and melanocortin 1 receptor (alpha melanocyte stimulating hormone receptor) and melanocortin receptor 1)	300	170/295(57%)	3/295 (1%)	188/297 (63%)	2/297 (0%)	272/300 (90%)	1/300 (0%)
127.	BAG38430 (melanocortin 1 receptor, matched with melanocortin 1 receptor and	300	170/295(57%)	3/295 (1%)	188/297 (63%)	2/297 (0%)	272/300 (90%)	1/300 (0%)

	melanocortin 1 receptor (alpha melanocyte stimulating hormone receptor) and melanocortin receptor 1)							
128.	BAG38431 (melanocortin 1 receptor, matched with melanocortin 1 receptor and melanocortin 1 receptor (alpha melanocyte stimulating hormone receptor) and melanocortin receptor 1)	300	170/295(57%)	3/295 (1%)	188/297 (63%)	2/297 (0%)	272/300 (90%)	1/300 (0%)
129.	BAG38432 (melanocortin 1 receptor, matched with melanocortin 1 receptor and melanocortin 1 receptor (alpha melanocyte stimulating hormone receptor) and melanocortin receptor 1)	300	170/295(57%)	3/295 (1%)	188/297 (63%)	2/297 (0%)	272/300 (90%)	1/300 (0%)
130.	BAG38433 (melanocortin 1 receptor, matched with melanocortin 1 receptor and melanocortin 1 receptor (alpha melanocyte stimulating hormone receptor) and melanocortin receptor 1)	300	170/295(57%)	3/295 (1%)	188/297 (63%)	2/297 (0%)	272/300 (90%)	1/300 (0%)
131.	BAG38434 (melanocortin 1 receptor, matched with melanocortin 1 receptor and melanocortin 1 receptor (alpha melanocyte stimulating hormone receptor) and melanocortin receptor 1)	300	170/295(57%)	3/295 (1%)	188/297 (63%)	2/297 (0%)	272/300 (90%)	1/300 (0%)

132.	BAG38435 (melanocortin 1 receptor, matched with melanocortin 1 receptor and melanocortin 1 receptor (alpha melanocyte stimulating hormone receptor) and melanocortin receptor 1)	300	170/295(57%)	3/295 (1%)	188/297 (63%)	2/297 (0%)	272/300 (90%)	1/300 (0%)
133.	BAG38436 (melanocortin 1 receptor, matched with melanocortin 1 receptor and melanocortin 1 receptor (alpha melanocyte stimulating hormone receptor) and melanocortin receptor 1)	300	170/295(57%)	3/295 (1%)	188/297 (63%)	2/297 (0%)	272/300 (90%)	1/300 (0%)
134.	BAG38437 (melanocortin 1 receptor, matched with melanocortin 1 receptor and melanocortin 1 receptor (alpha melanocyte stimulating hormone receptor) and melanocortin receptor 1)	300	170/295(57%)	3/295 (1%)	188/297 (63%)	2/297 (0%)	272/300 (90%)	1/300 (0%)
135.	BAG38438 (melanocortin 1 receptor, matched with melanocortin 1 receptor and melanocortin 1 receptor (alpha melanocyte stimulating hormone receptor) and melanocortin receptor 1)	300	170/295(57%)	3/295 (1%)	188/297 (63%)	2/297 (0%)	272/300 (90%)	1/300 (0%)
136.	BAG38469 (melanocortin 4 receptor, matched with melanocortin 4 receptor variant and melanocortin 4 receptor and melanocortin 4 receptor)	322	219/302(72%)	7/302 (2%)	219/316 (69%)	2/316 (0%)	302/323 (93%)	1/323 (0%)

137.	BAG38470 (melanocortin 4 receptor, matched with melanocortin 4 receptor and melanocortin 4 receptor)	322	219/302(72%)	7/302 (2%)	222/323 (68%)	7/323 (2%)	303/323 (93%)	1/323 (0%)
138.	BAG38471 (melanocortin 4 receptor, matched with melanocortin 4 receptor and melanocortin 4 receptor)	322	219/302(72%)	7/302 (2%)	222/323 (68%)	7/323 (2%)	303/323 (93%)	1/323 (0%)
139.	BAG38472 (melanocortin 4 receptor, matched with melanocortin 4 receptor and melanocortin 4 receptor)	322	219/302(72%)	7/302 (2%)	222/323 (68%)	7/323 (2%)	303/323 (93%)	1/323 (0%)
140.	BAG38473 (melanocortin 4 receptor, matched with melanocortin 4 receptor and melanocortin 4 receptor)	322	219/302(72%)	7/302 (2%)	222/323 (68%)	7/323 (2%)	303/323 (93%)	1/323 (0%)
141.	BAG38474 (melanocortin 4 receptor, matched with melanocortin 4 receptor and melanocortin 4 receptor)	322	219/302(72%)	7/302 (2%)	222/323 (68%)	7/323 (2%)	303/323 (93%)	1/323 (0%)
142.	BAG38475 (melanocortin 4 receptor, matched with melanocortin 4 receptor and melanocortin 4 receptor)	322	219/302(72%)	7/302 (2%)	222/323 (68%)	7/323 (2%)	303/323 (93%)	1/323 (0%)
143.	BAG38476 (melanocortin 4 receptor, matched with melanocortin 4 receptor and melanocortin 4 receptor)	322	219/302(72%)	7/302 (2%)	222/323 (68%)	7/323 (2%)	303/323 (93%)	1/323 (0%)
144.	BAG38477 (melanocortin 4 receptor, matched with melanocortin 4 receptor and melanocortin 4 receptor)	322	219/302(72%)	7/302 (2%)	222/323 (68%)	7/323 (2%)	303/323 (93%)	1/323 (0%)
145.	BAG38478 (melanocortin 4 receptor, matched with melanocortin 4 receptor and	322	219/302(72%)	7/302 (2%)	222/323 (68%)	7/323 (2%)	303/323 (93%)	1/323 (0%)

	melanocortin 4 receptor)							
146.	BAG38479 (melanocortin 4 receptor, matched with melanocortin 4 receptor and melanocortin 4 receptor)	322	219/302(72%)	7/302 (2%)	222/323 (68%)	7/323 (2%)	303/323 (93%)	1/323 (0%)
147.	BAG38480 (melanocortin 4 receptor, matched with melanocortin 4 receptor and melanocortin 4 receptor)	322	219/302(72%)	7/302 (2%)	222/323 (68%)	7/323 (2%)	303/323 (93%)	1/323 (0%)
148.	BAG38481 (melanocortin 4 receptor, matched with melanocortin 4 receptor and melanocortin 4 receptor)	322	219/302(72%)	7/302 (2%)	222/323 (68%)	7/323 (2%)	303/323 (93%)	1/323 (0%)
149.	BAG38482 (melanocortin 4 receptor, matched with melanocortin 4 receptor and melanocortin 4 receptor)	322	219/302(72%)	7/302 (2%)	222/323 (68%)	7/323 (2%)	303/323 (93%)	1/323 (0%)
150.	BAG38483 (melanocortin 4 receptor, matched with melanocortin 4 receptor and melanocortin 4 receptor)	322	219/302(72%)	7/302 (2%)	222/323 (68%)	7/323 (2%)	303/323 (93%)	1/323 (0%)
151.	BAG38484 (melanocortin 4 receptor, matched with melanocortin 4 receptor and melanocortin 4 receptor)	322	219/302(72%)	7/302 (2%)	222/323 (68%)	7/323 (2%)	303/323 (93%)	1/323 (0%)
152.	BAG38485 (melanocortin 4 receptor, matched with melanocortin 4 receptor and melanocortin 4 receptor)	322	219/302(72%)	7/302 (2%)	222/323 (68%)	7/323 (2%)	303/323 (93%)	1/323 (0%)
153.	BAG38486 (melanocortin 4 receptor, matched with melanocortin 4 receptor and melanocortin 4 receptor)	322	219/302(72%)	7/302 (2%)	222/323 (68%)	7/323 (2%)	303/323 (93%)	1/323 (0%)
154.	BAG38487 (melanocortin 4 receptor, matched with	322	219/302(72%)	7/302 (2%)	222/323 (68%)	7/323 (2%)	303/323 (93%)	1/323 (0%)

	melanocortin 4 receptor and melanocortin 4 receptor)							
155.	BAG38488 (melanocortin 4 receptor, matched with melanocortin 4 receptor and melanocortin 4 receptor)	322	219/302(72%)	7/302 (2%)	222/323 (68%)	7/323 (2%)	303/323 (93%)	1/323 (0%)
156.	BAG38489 (melanocortin 4 receptor, matched with melanocortin 4 receptor and melanocortin 4 receptor)	322	219/302(72%)	7/302 (2%)	222/323 (68%)	7/323 (2%)	303/323 (93%)	1/323 (0%)
157.	BAG38490 (melanocortin 4 receptor, matched with melanocortin 4 receptor and melanocortin 4 receptor)	322	219/302(72%)	7/302 (2%)	222/323 (68%)	7/323 (2%)	303/323 (93%)	1/323 (0%)
158.	BAG38491 (melanocortin 4 receptor, matched with melanocortin 4 receptor and melanocortin 4 receptor)	322	219/302(72%)	7/302 (2%)	222/323 (68%)	7/323 (2%)	303/323 (93%)	1/323 (0%)
159.	BAG38492 (melanocortin 4 receptor, matched with melanocortin 4 receptor and melanocortin 4 receptor)	322	219/302(72%)	7/302 (2%)	222/323 (68%)	7/323 (2%)	303/323 (93%)	1/323 (0%)
160.	CAA56455 (dopamine receptor, matched with dopamine receptor D1 and unnamed protein product)	459	317/451(70%)	18/451 (3%)	335/457 (73%)	18/457 (3%)	427/458 (93%)	1/458 (0%)
161.	CAA56456 (dopamine receptor, matched with dopamine receptor D2 and unnamed protein product)	463	309/472(65%)	36/472 (7%)	311/461 (67%)	35/461 (7%)	412/437 (94%)	1/437 (0%)
162.	CAA56457 (dopamine receptor, matched with dopamine receptor D5 and dopamine D1C receptor and unnamed protein product)	463	262/448(58%)	37/448 (8%)	250/427 (58%)	27/427 (6%)	351/374 (93%)	3/374 (0%)

163.	CAA58745 (serotonin receptor, matched with 5-hydroxytryptamine (serotonin) receptor 1D and unnamed protein product)	379	256/354(72%)	8/354 (2%)	267/379 (70%)	13/379 (3%)	236/369 (63%)	13/369 (3%)
164.	CAA64174 (cannabinoid receptor type 1A, matched with cannabinoid receptor 1 (brain) and unnamed protein product)	468	344/474(72%)	8/474 (1%)	357/473 (75%)	5/473 (1%)	448/468 (95%)	0/468 (0%)
165.	CAA64175 (cannabinoid receptor type 1B, matched with cannabinoid receptor 1 (brain) and unnamed protein receptor)	470	288/481(59%)	20/481 (4%)	297/482 (61%)	21/482 (4%)	434/474 (91%)	4/474 (0%
166.	CAA65175 (serotonin receptor, matched with 5-hydroxytryptamine (serotonin) receptor 1A and unnamed protein receptor)	423	298/408(73%)	13/408 (3%)	306/404 (75%)	12/404 (2%)	403/422 (95%)	0/422 (0%)
167.	CAA65176 (serotonin receptor, matched with 5-hydroxytryptamine (serotonin) receptor 1A and unnamed protein product)	416	267/423(63%)	18/423 (4%)	289/395 (73%)	12/395 (3%)	383/415 (92%)	0/415 (0%)
168.	CAC82587 (vasoactive intestinal peptide receptor, matched with vasoactive intestinal peptide receptor 1 and unnamed protein product)	419	232/422(54%)	5/422 (1%)	266/423 (62%)	8/423 (1%)	333/421 (79%)	37/421 (8%)
169.	CAC82588 (vasoactive intestinal peptide receptor 1 A, matched with vasoactive intestinal peptide receptor 1 and unnamed protein	419	237/415(57%)	8/415 (1%)	259/414 (62%)	7/414 (1%)	361/394 (91%)	11/394 (2%)

	product)							
170.	CAC82589 (growth hormone releasing hormone-like peptide receptor, matched with adenylate cyclase activating polypeptide 1 (pituitary) receptor type I and growth-hormone releasing hormone-like peptide receptor and unnamed protein product)	438	193/397(48%)	33/397 (8%)	217/378 (57%)	6/378 (1%)	335/417 (80%	42/417 (10%)
171.	CAC82924 (corticotrophin releasing factor receptor, matched with corticotropin releasing hormone receptor 1 and unnamed protein product)	434	324/383(84%)	0/383 (0%)	327/393 (83%)	1/393 (0%)	299/365 (81%)	49/365 (13%)
172.	CAC83860 (vasoactive intestinal peptide receptor, matched with vasoactive intestinal peptide receptor 2 and unnamed protein product)	425	212/408(51%)	4/408 (0%)	224/409 (54%)	6/409 (1%)	324/438 (73%)	42/438 (9%)
173.	CAC83861 (vasoactive intestinal peptide receptor, matched with vasoactive intestinal peptide receptor 2 and unnamed protein product)	414	245/417(58%)	6/417 (1%)	257/419 (61%)	6/419 (1%)	276/371 (74%)	61/371 (16%)
174.	CAC87880 (alpha2 a1 adrenergic receptor, matched with adrenergic, alpha-2A-, receptor and alpha-2B adrenergic receptor and unnamed protein product)	388	243/452(53%)	70/452(15%)	166/310 (53%)	33/310(10%)	364/388 (93%)	2/388 (0%)
175.	CAC87881 (alpha2 c1 adrenergic receptor, matched	447	242/443(54%)	23/443 (5%)	172/231 (74%)	12/231 (5%)	424/447 (94%)	1/447 (0%)

	with adrenergic, alpha-2A-, receptor and adrenergic, alpha-2C-, receptor and unnamed protein product)							
176.	CAC87882 (alpha2 b1 adrenergic receptor, matched with adrenergic, alpha-2A-, receptor and adrenergic, alpha-2C-, receptor and unnamed protein product)	510	232/478(48%)	60/478(12%)	124/183 (67%)	2/183 (1%)	432/525 (82%)	15/525 (2%)
177.	CAC87883 (alpha2 b2 adrenergic receptor, matched with adrenergic, alpha-2B-, receptor and adrenergic, alpha-2C-, receptor and unnamed protein product)	465	153/269(56%)	28/269(10%)	133/186 (71%)	5/186 (2%)	426/456 (93%)	0/456 (0%)
178.	CAC87884 (alpha2 a2 adrenergic receptor, matched with adrenergic, alpha-2A-, receptor and adrenergic, alpha-2C-, receptor and unnamed protein product)	417	273/429(63%)	32/429 (7%)	153/242 (63%)	15/242 (6%)	356/406 (87%)	4/406 (0%)
179.	CAC87885 (alpha2 c2 adrenergic receptor, matched with adrenergic, alpha-2C-, receptor and adrenergic, alpha-2C-, receptor and unnamed protein product)	420	264/420(62%)	24/420 (5%)	167/223 (74%)	5/223 (2%)	344/420 (81%)	17/420 (4%)
180.	CAC87886 (alpha2 d1 adrenergic receptor, matched with adrenergic, alpha-2C-, receptor and unnamed protein product)	420	201/374(53%)	22/374 (5%)	131/189 (69%)	6/189 (3%)	272/434 (62%)	56/434 (12%)
181.	CAC87887 (alpha2 d2 adrenergic receptor, matched with adrenergic, alpha-2A-, receptor and adrenergic,	471	207/438(47%)	84/438(19%)	99/179 (55%)	14/179 (7%)	380/441 (86%)	20/441 (4%)

	alpha-2B-, receptor and unnamed protein product)							
182.	CAD35690 (pituitary adenylate-cyclase activating polypeptide receptor 1A, matched with adenylate cyclase activating polypeptide 1 (pituitary) receptor type I and vasoactive intestinal peptide receptor 1 and unnamed protein product)	444	316/424(74%)	29/424 (6%)	214/397 (53%)	12/397 (3%)	304/316 (96%)	0/316 (0%)
183.	CAD38842 (pituitary adenylate cyclase-activating polypeptide 1B, matched with adenylate cyclase activating polypeptide 1 (pituitary) receptor type I and pituitary adenylate cyclase-activating polypeptide type I receptor precursor short form and unnamed protein product)	444	316/422(74%)	25/422 (5%)	323/425 (76%)	28/425 (6%)	340/448 (75%)	81/448 (18%)
184.	CAD67555 (parathyroid hormone receptor 3, matched with parathyroid hormone 1 receptor and unnamed protein product)	559	279/474(58%)	62/474(13%)	282/417 (67%)	13/417 (3%)	353/402 (87%)	11/402 (2%)
185.	CAD68048 (parathyroid hormone receptor, matched with parathyroid hormone 2 receptor and parathyroid hormone 1 receptor and unnamed protein product)	559	314/544(57%)	19/544 (3%)	287/530 (54%)	27/530 (5%)	274/306 (89%)	2/306 (0%)
186.	CAD79707 (parathyroid hormone receptor 1, matched with parathyroid hormone 1 receptor and unnamed protein product)	532	349/583(59%)	71/583(12%)	399/515 (77%)	4/515 (0%)	433/545 (79%)	79/545 (14%)

187.	DAA06178 (luteinizing hormone receptor, matched with luteinizing hormone/choriogonadotropin receptor)	691	318/585(54%)	19/585(3%)	348/643 (54%)	39/643(6%)	504/572 (88%)	2/572 (0%)
188.	FAA00372 (calcitonin receptor, matched with calcitonin receptor-like and calcitonin receptor and TPA calcitonin receptor)	794	287/451(63%)	11/451 (2%)	329/402 (81%)	2/402 (0%)	703/799 (87%)	8/799 (1%)
189.	NP_001027732 (melanocortin 4 receptor, matched with melanocortin 4 receptor and melanocortin 4 receptor)	322	219/302(72%)	7/302 (2%)	222/323 (68%)	7/323 (2%)	303/323 (93%)	1/323 (0%)
190.	NP_001027778 (multiple tissue opsin, matched with opsin 3 and similar to multiple tissue opsin and unnamed protein product)	402	103/254(40%)	9/254 (3%)	178/281 (63%)	1/281 (0%)	206/236 (87%)	2/236 (0%)
191.	NP_001027835 (G protein-coupled receptor 119, matched with G protein-coupled receptor 119 and unnamed protein product)	393	131/315(41%)	30/315 (9%)	115/317 (36%)	39/317(12%)	249/337 (73%)	27/337 (8%)
192.	NP_001027836 (G protein-coupled receptor 135, matched with G protein-coupled receptor 135 and unnamed protein product)	444	200/358(55%)	13/358 (3%)	177/299 (59%)	10/299 (3%)	238/257 (92%)	0/257 (0%)
193.	NP_001027859 (G protein-coupled receptor 100, matched with relaxin/insulin-like family peptide receptor 3 and unnamed protein product)	408	231/386(59%)	35/386 (9%)	244/376 (64%)	22/376 (5%)	314/349 (89%)	0/349 (0%)
194.	NP_001027936 (melanocortin 2 receptor, matched with	301	144/295(48%)	12/295 (4%)	147/298 (49%)	8/298 (2%)	266/299	0/299

	adrenocorticotropic hormone receptor, ACTH receptor and melanocortin 2 receptor and melanocortin 2 receptor)						(88%)	(0%)
195.	NP_001027937 (melanocortin 5 receptor, matched with melanocortin 5 receptor and melanocortin 5 receptor)	340	227/294(77%)	0/294 (0%)	246/323 (76%)	7/323 (2%)	304/340 (89%)	0/340 (0%)
196.	NP_001028884 (green opsin, matched with rhodopsin and rhodopsin (opsin 2, rod pigment) (retinitis pigmentosa 4, autosomal dominant) and green-sensitive pigment)	352	229/345(66%)	2/345 (0%)	237/342 (69%)	4/342 (1%)	330/352 (93%)	1/352 (0%)
197.	NP_001029021 (rod-like opsin, matched with rhodopsin and unnamed protein product)	353	262/332(78%)	0/332 (0%)	265/332 (79%)	0/332 (0%)	340/353 (96%)	0/353 (0%)
198.	NP_001035912 (progestin and adipoQ receptor family member VII, matched with progestin and adipoQ receptor family member VIII and progestin and adipoQ receptor family member VII and unnamed protein product)	352	175/354(49%)	2/354 (0%)	184/324 (56%)	6/324 (1%)	342/350 (97%)	0/350 (0%)
199.	NP_001072090 (interleukin 8 receptor II, matched with interleukin 8 receptor, alpha and interleukin 8 receptor, beta and unnamed protein product)	359	141/336(41%)	23/336 (6%)	102/236 (43%)	2/236 (0%)	158/308 (51%)	3/308 (0%)
200.	NP_001072097 (taste receptor, type 1, member 3, matched with taste receptor,	853	306/808(37%)	17/808 (2%)	309/806 (38%)	16/806 (1%)	678/829 (81%)	0/829 (0%)

	type 1, member 3 and unnamed protein product)							
201.	NP_001072099 (rod opsin, matched with rhodopsin and unnamed protein product)	353	262/327(80%)	0/327 (0%)	265/327 (81%)	0/327 (0%)	328/346 (94%)	0/346 (0%)
202.	NP_001072110 (interleukin 8 receptor I transcript 1, matched with interleukin 8 receptor, alpha and interleukin 8 receptor, beta and unnamed protein product)	352	111/263(42%)	3/263 (1%)	111/263 (42%)	3/263 (1%)	262/318 (82%)	4/318 (1%)
203.	NP_001091093 (interleukin 8 receptor I transcript 2, matched with interleukin 8 receptor, alpha and interleukin 8 receptor, beta and unnamed protein product)	352	111/263(42%)	3/263 (1%)	111/263 (42%)	3/263 (1%)	262/318 (82%)	4/318 (1%)
204.	NP_001092114 (flg-Hepta protein, matched with G protein-coupled receptor 116 and unnamed protein product)	1678	276/801(34%)	89/801(11%)	257/708 (36%)	73/708(10%)	150/312 (48%)	32/312 (10%)
205.	NP_001092117 (RFamide-related peptide receptor, matched with neuropeptide FF receptor 2 and neuropeptide FF receptor 1 and unnamed protein product)	474	196/406(48%)	31/406 (7%)	204/364 (56%)	15/364 (4%)	294/339 (86%)	0/339 (0%)
206.	NP_001092118 (neuropeptide FF receptor-1 NPFF2-1, matched with neuropeptide FF receptor 2 and unnamed protein product)	426	262/403(65%)	1/403 (0%)	298/416 (71%)	2/416 (0%)	324/389 (83%)	43/389 (11%)

207.	NP_001092119 (neuropeptide FF receptor-2 NPFF2-2, matched with neuropeptide FF receptor 2 and unnamed protein product)	453	245/422(58%)	41/422 (9%)	259/416 (62%)	29/416 (6%)	316/398 (79%)	42/398 (10%)
208.	NP_001092135 (endothelin receptor type A, matched with endothelin receptor type A and unnamed protein product)	468	238/380(62%)	26/380 (6%)	246/403 (61%)	32/403 (7%)	289/384 (75%)	32/384 (8%)
209.	NP_001098073 (neuropeptide Y/peptide YY receptor, matched with neuropeptide Y receptor Y1 and pancreatic polypeptide receptor 1 and unnamed protein product)	406	169/354(47%)	28/354 (7%)	178/363 (49%)	26/363 (7%)	278/304 (91%)	21/304 (6%)
210.	NP_001098074 (neuropeptide Y/peptide YY receptor Npy8br, matched with neuropeptide Y receptor Y1 and NPY receptor Y6 and unnamed protein product)	364	174/337(51%)	3/337 (0%)	184/346 (53%)	3/346 (0%)	305/345 (88%)	0/345 (0%)
211.	NP_001098075 (neuropeptide Y/peptide YY receptor Npy4r, matched with neuropeptide Y receptor Y1 and pancreatic polypeptide receptor 1 and unnamed protein product)	411	172/368(46%)	11/368 (2%)	190/374 (50%)	14/374 (3%)	263/291 (90%)	4/291 (1%)
212.	NP_001098685 (pituitary adenylate-cyclase activating polypeptide receptor 1A, matched with adenylate cyclase activating polypeptide 1 (pituitary) receptor type I and unnamed protein	444	316/424(74%)	29/424 (6%)	332/435 (76%)	30/435 (6%)	304/316 (96%)	0/316 (0%)

	product)							
213.	NP_001098686 (pituitary adenylate cyclase-activating polypeptide 1B, matched with adenylate cyclase activating polypeptide 1 (pituitary) receptor type I and unnamed protein product)	444	316/422(74%)	25/422 (5%)	323/425 (76%)	28/425 (6%)	340/448 (75%)	81/448 (18%)
214.	NP_001098687 (taste receptor, type 1, member 2a, matched with taste receptor, type 1, member 1 and unnamed protein product)	824	291/833(34%)	26/833 (3%)	222/667 (33%)	30/667 (4%)	671/838 (80%)	23/838 (2%)
215.	NP_001098688 (taste receptor, type 1, member 2b, matched with taste receptor, type 1, member 1 and unnamed protein product)	816	304/821(37%)	40/821 (4%)	242/668 (36%)	35/668 (5%)	541/738 (73%)	1/738 (0%)
216.	NP_001098689 (calcitonin receptor, matched with calcitonin receptor-like and calcitonin receptor and TPA calcitonin receptor)	794	287/451(63%)	11/451 (2%)	329/402 (81%)	2/402 (0%)	703/799 (87%)	8/799 (1%)
217.	NP_001098693 (neuropeptide Y receptor 2, matched with neuropeptide Y receptor Y2 and unnamed protein product)	396	201/306(65%)	5/306 (1%)	203/316 (64%)	7/316 (2%)	309/339 (91%)	0/339 (0%)
218.	NP_001098695 (neuropeptide Y receptor Y7, matched with neuropeptide Y receptor Y2 and neuropeptide Y7 receptor and unnamed protein product)	369	202/362(55%)	7/362 (1%)	240/339 (70%)	5/339 (1%)	172/301 (57%)	7/301 (2%)
219.	NP_001098703 (G protein-coupled receptor 155, matched with G protein-	843	444/827(53%)	44/827 (5%)	448/819 (54%)	36/819 (4%)	559/733 (76%)	111/733 (15%)

	coupled receptor 155 and unnamed protein product)							
220.	NP_001098705 (G-protein coupled receptor 39-1, matched with G protein-coupled receptor 39 and unnamed protein product)	417	197/376(52%)	24/376 (6%)	195/368 (52%)	22/368 (5%)	242/244 (99%)	0/244 (0%)
221.	NP_001127927 (melanocortin 1 receptor, matched with melanocortin 1 receptor and melanocortin 1 receptor (alpha melanocyte stimulating hormone receptor) and melanocortin I receptor)	300	170/295(57%)	3/295 (1%)	188/297 (63%)	2/297 (0%)	272/300 (90%)	1/300 (0%)
222.	O42179 (Somatostatin-like receptor, matched with somatostatin receptor 5 and unnamed protein product)	289	133/229(58%)	24/229(10%)	138/238 (57%)	35/238(14%)	185/227 (81%)	23/227 (10%)
223.	O42384 (Serotonin receptor 1A-beta, matched with 5-hydroxytryptamine (serotonin) receptor 1A and 5-hydroxytryptamine (serotonin) receptor 1A and unnamed protein product)	416	267/423(63%)	18/423 (4%)	289/395 (73%)	12/395 (3%)	383/415 (92%)	0/415 (0%)
224.	O42385 (Serotonin receptor 1A-alpha, matched with 5-hydroxytryptamine (serotonin) receptor 1A and unnamed protein product)	423	298/408(73%)	13/408 (3%)	306/404 (75%)	12/404 (2%)	403/422 (95%)	0/422 (0%)
225.	P53452 (D(1)-like dopamine receptor, matched with dopamine receptor D1 and unnamed protein product)	459	317/451(70%)	18/451 (3%)	335/457 (73%)	18/457 (3%)	427/458 (93%)	1/458 (0%)
226.	P53453 (D(2)-like dopamine receptor, matched with	463	309/472(65%)	36/472 (7%)	311/461 (67%)	35/461 (7%)	412/437	1/437

	dopamine receptor D2 and unnamed protein product)						(94%)	(0%)
227.	P53454 (D(5)-like dopamine receptor, matched with dopamine receptor D5 and dopamine D1C receptor and unnamed protein product)	463	262/448(58%)	37/448 (8%)	250/427 (58%)	27/427 (6%)	351/374 (93%)	3/374 (0%)
228.	P79748 (Serotonin receptor 1D, matched with 5-hydroxytryptamine (serotonin) receptor 1D and unnamed protein product)	379	256/354(72%)	8/354 (2%)	267/379 (70%)	13/379 (3%)	236/369 (63%)	13/369 (3%)
229.	Q9PUQ8 (Sphingosine 1-phosphate receptor 3, matched with sphingosine-1-phosphate receptor 3 and endothelial differentiation, sphingolipid G-protein-coupled receptor, 3 and unnamed protein product)	384	214/305(70%)	3/305 (0%)	220/312 (70%)	4/312 (1%)	295/329 (89%),	0/329 (0%)
230.	Q98894 (Cannabinoid receptor type 1A, matched with cannabinoid receptor 1 (brain) and unnamed protein product)	468	344/474 (72%	8/474 (1%)	357/473 (75%)	5/473 (1%)	448/468 (95%),	0/468 (0%)
231.	Q98895 (Cannabinoid receptor type 1B, matched with cannabinoid receptor 1 (brain) and unnamed protein product)	470	288/481(59%)	20/481 (4%)	297/482 (61%)	21/482 (4%)	434/474 (91%)	4/474 (0%)
**II.**	**UNIPROT Sequences**							
232.	O73635 (Calcium2+ sensing receptor, matched with calcium-sensing receptor and unnamed protein product)	940	669/890(75%)	16/890 (1%)	672/890 (75%)	15/890 (1%)	829/907 (91%)	38/907 (4%)

233.	O73636 (Pheromone receptor, matched with calcium-sensing receptor and unnamed protein product)	868	340/841(40%)	42/841 (4%)	344/844 (40%)	49/844 (5%)	371/763 (48%)	27/763 (3%)
234.	O73637 (Pheromone receptor, matched with calcium-sensing receptor and unnamed protein product)	864	360/847 (42%	36/847 (4%)	361/846 (42%)	35/846 (4%)	640/770 (83%)	0/770 (0%)
235.	O73638 (Pheromone receptor, matched with calcium-sensing receptor and unnamed protein product)	856	352/843(41%)	38/843 (4%)	360/875 (41%)	46/875 (5%)	426/478 (89%)	0/478 (0%)
236.	O73639 (Pheromone receptor, matched with calcium-sensing receptor and unnamed protein product)	880	372/857(43%)	32/857 (3%)	367/846 (43%)	31/846 (3%)	709/864 (82%)	61/864 (7%)
237.	O73640 (Pheromone receptor, matched with calcium-sensing receptor and unnamed protein product)	875	324/851(38%)	38/851 (4%)	325/850 (38%)	37/850 (4%)	467/647 (72%)	26/647 (4%)
238.	O73641 (Metabotropic glutamate receptor 1 homologue (Fragment), matched with glutamate receptor, metabotropic 1 and unnamed protein product)	156	137/156(87%)	0/156 (0%)	138/156 (88%)	0/156 (0%)	150/156 (96%)	0/156 (0%)
239.	O73642 (Metabotropic glutamate receptor 2 homologue (Fragment), matched with glutamate receptor, metabotropic 2 and glutamate receptor, metabotropic 3 and unnamed protein product)	106	75/106 (70%)	0/106 (0%)	71/106 (66%)	0/106 (0%)	100/106 (94%)	0/106 (0%)
240.	O73643 (Metabotropic glutamate receptor 7 homologue (Fragment),	161	122/161(75%)	0/161 (0%)	123/161 (76%)	0/161 (0%)	156/172 (90%)	11/172 (6%)

	matched with glutamate receptor, metabotropic 6 and glutamate receptor, metabotropic 7 and unnamed protein product)							
241.	O73644 (Metabotropic glutamate receptor 8 homologue (Fragment), matched with glutamate receptor, metabotropic 8 and unnamed protein product)	111	84/111 (75%)	0/111 (0%)	82/111 (73%)	0/111 (0%)	107/111 (96%)	0/111 (0%)
242.	O73645 (Pheromone receptor (Fragment), matched with calcium-sensing receptor and unnamed protein product)	250	132/250(52%)	0/250 (0%)	132/250 (52%)	0/250 (0%)	195/211 (92%)	0/211 (0%)
243.	O73646 (Pheromone receptor (Fragment), matched with calcium-sensing receptor and unnamed protein product)	250	127/232(54%)	0/232 (0%)	131/234 (55%)	4/234 (1%)	224/250 (89%)	0/250 (0%)
244.	O73647 (Pheromone receptor (Fragment), matched with calcium-sensing receptor and unnamed protein product)	250	135/249(54%)	0/249 (0%)	136/249 (54%)	0/249 (0%)	204/250 (81%)	0/250 (0%)
245.	O73648 (Pheromone receptor (Fragment), matched with calcium-sensing receptor and unnamed protein product)	239	116/226(51%)	0/226 (0%)	116/226 (51%)	0/226 (0%)	175/226 (77%)	0/226 (0%)
246.	O73649 (Pheromone receptor (Fragment), matched with calcium-sensing receptor and unnamed protein product)	250	136/251(54%)	2/251 (0%)	135/251 (53%)	2/251 (0%)	232/250 (92%)	0/250 (0%)
247.	O73650 (Pheromone receptor (Fragment), matched with calcium-sensing receptor and unnamed protein product)	250	127/234(54%)	0/234 (0%)	128/234 (54%)	0/234 (0%)	201/249 (80%)	0/249 (0%)

248.	O73651 (Pheromone receptor (Fragment), matched with calcium-sensing receptor and unnamed protein product)	250	124/230(53%)	0/230 (0%)	125/230 (54%)	0/230 (0%)	196/250 (78%)	0/250 (0%)
249.	O73652 (Pheromone receptor (Fragment), matched with calcium-sensing receptor and unnamed protein product)	251	127/251(50%)	1/251 (0%)	125/251 (49%)	1/251 (0%)	224/251 (89%)	0/251 (0%)
250.	O73653 (Pheromone receptor (Fragment), matched with calcium-sensing receptor and unnamed protein product)	251	131/251(52%)	1/251 (0%)	130/251 (51%)	1/251 (0%)	218/251 (86%)	0/251 (0%)
251.	O73654 (Pheromone receptor (Fragment), matched with calcium-sensing receptor and unnamed protein product)	251	130/251(51%)	1/251 (0%)	128/251 (50%)	1/251 (0%)	229/251 (91%)	0/251 (0%)
252.	O73655 (Pheromone receptor (Fragment), matched with calcium-sensing receptor and unnamed protein product)	251	133/251(52%)	1/251 (0%)	116/227 (51%)	1/227 (0%)	225/251 (89%)	0/251 (0%)
253.	O73656 (Pheromone receptor (Fragment), matched with calcium-sensing receptor and unnamed protein product)	251	133/251(52%)	1/251 (0%)	132/251 (52%)	1/251 (0%)	221/251 (88%)	0/251 (0%)
254.	O73657 (Pheromone receptor (Fragment), matched with calcium-sensing receptor and unnamed protein product)	251	120/226(53%)	1/226 (0%)	120/227 (52%)	1/227 (0%)	202/220 (91%)	1/220 (0%)
255.	Q2MHJ8 (Taste receptor, type 1, member 3, matched with taste receptor, type 1, member 3 and unnamed protein product)	853	306/808(37%)	17/808 (2%)	309/806 (38%)	16/806 (1%)	678/829 (81%)	0/829 (0%)
256.	Q2MHJ9 (Taste receptor, type 1, member 2b, matched with taste receptor, type 1,	816	304/821(37%)	40/821 (4%)	242/668 (36%)	35/668 (5%)	541/738 (73%)	1/738 (0%)

	member 1 and unnamed protein product)							
257.	Q2MHK0 (Taste receptor, type 1, member 2a, matched with taste receptor, type 1, member 1 and unnamed protein product)	824	291/833(34%)	26/833 (3%)	222/667 (33%)	30/667 (4%)	671/838 (80%)	23/838 (2%)
258.	Q2MHK1 (Taste receptor, type 1, member 1, matched with taste receptor, type 1, member 1 and unnamed protein product)	845	345/810(42%)	13/810 (1%)	285/685 (41%)	13/685 (1%)	715/841 (85%)	0/841 (0%)
259.	Q2UZQ8 (Parathyroid hormone receptor 1, matched with parathyroid hormone 1 receptor and unnamed protein product)	532	349/583(59%)	71/583(12%)	399/515 (77%)	4/515 (0%)	433/545 (79%)	79/545 (14%)
260.	Q2UZQ9 (Parathyroid hormone receptor, matched with parathyroid hormone 2 receptor and parathyroid hormone 1 receptor and unnamed protein product)	559	314/544(57%)	19/544 (3%)	287/530 (54%)	27/530 (5%)	274/306 (89%)	2/306 (0%)
261.	Q2UZR0 (Parathyroid hormone receptor 3, matched with parathyroid hormone 1 receptor and unnamed protein product)	559	279/474(58%)	62/474(13%)	282/417 (67%)	13/417 (3%)	353/402 (87%)	11/402 (2%)
262.	Q05KN3 (Neuropeptide FF receptor-1 NPFF2-1, matched with neuropeptide FF receptor 2 and unnamed protein product)	426	262/403(65%)	1/403 (0%)	298/416 (71%)	2/416 (0%)	324/389 (83%)	43/389 (11%)
263.	Q05KN4 (RFamide-related peptide receptor, matched with neuropeptide FF receptor 2 and neuropeptide	474	196/406(48%)	31/406 (7%)	204/364 (56%)	15/364 (4%)	294/339 (86%)	0/339 (0%)

	FF receptor 1 and unnamed protein product)							
264.	Q5WML0 (Pituitary adenylate cyclase-activating polypeptide 1B, matched with adenylate cyclase activating polypeptide 1 (pituitary) receptor type I and unnamed protein product)	444	316/422(74%)	25/422 (5%)	323/425 (76%)	28/425 (6%)	340/448 (75%)	81/448 (18%)
265.	Q5WML1 (Pituitary adenylate-cyclase activating polypeptide receptor 1A, matched with adenylate cyclase activating polypeptide 1 (pituitary) receptor type I and unnamed protein product)	444	316/424(74%)	29/424 (6%)	332/435 (76%)	30/435 (6%)	304/316 (96%)	0/316 (0%)
266.	Q7T2L0 (G protein-coupled receptor 135, matched with G protein-coupled receptor 135 and unnamed protein product)	444	200/358(55%)	13/358 (3%)	177/299 (59%)	10/299 (3%)	238/257 (92%)	0/257 (0%)
267.	Q7T2L1 (G protein-coupled receptor 142b (Fragment), matched with G protein-coupled receptor 139 and unnamed protein product)	383	245/377(64%)	25/377 (6%)	243/380 (63%)	33/380 (8%)	311/328 (94%)	1/328 (0%)
268.	Q7T2L2 (G protein-coupled receptor 142a (Fragment), matched with G protein-coupled receptor 142 and kinesin family member 19 and unnamed protein product)	371	172/296(58%)	11/296 (3%)	180/308 (58%)	19/308 (6%)	293/315 (93%)	6/315 (1%)
269.	Q7T2L3 (G protein-coupled receptor 136 (Fragment), matched with opsin 5 and unnamed protein product)	259	182/313(58%)	56/313(17%)	173/316 (54%)	59/316(18%)	175/195 (89%)	7/195 (3%)

270.	Q7T2L4 (G protein-coupled receptor 119, matched with G protein-coupled receptor 119 and unnamed protein product)	393	131/315(41%)	30/315 (9%)	115/317 (36%)	39/317(12%)	249/337 (73%)	27/337 (8%)
271.	Q7T2L5 (G protein-coupled receptor 100, matched with relaxin/insulin-like family peptide receptor 3 and unnamed protein product)	408	231/386(59%)	35/386 (9%)	244/376 (64%)	22/376 (5%)	314/349 (89%)	0/349 (0%)
272.	Q8AXV3 (Vasoactive intestinal peptide receptor 1 A, matched with vasoactive intestinal peptide receptor 1 and unnamed protein product)	419	237/415(57%)	8/415 (1%)	259/414 (62%)	7/414 (1%)	361/394 (91%)	11/394 (2%)
273.	Q8AXV4 (Vasoactive intestinal peptide receptor, matched with vasoactive intestinal peptide receptor 1 and unnamed protein product)	419	232/422(54%)	5/422 (1%)	266/423 (62%)	8/423 (1%)	333/421 (79%)	37/421 (8%)
274.	Q19WU5 (Progestin and adipoQ receptor family member VII, matched with progestin and adipoQ receptor family member VIII and unnamed protein product)	352	175/354(49%)	2/354 (0%)	168/353 (47%)	1/353 (0%)	342/350 (97%)	0/350 (0%)
275.	Q802T6 (Vasoactive intestinal peptide receptor (Fragment), matched with vasoactive intestinal peptide receptor 2 and unnamed protein product)	414	245/417(58%)	6/417 (1%)	257/419 (61%)	6/419 (1%)	276/371 (74%)	61/371 (16%)
276.	Q802T7 (Vasoactive intestinal peptide receptor (Fragment), matched with vasoactive	425	212/408(51%)	4/408 (0%)	224/409 (54%)	6/409 (1%)	324/438 (73%)	42/438 (9%)

	intestinal peptide receptor 2 and unnamed protein product)							
**III.**	**Frizzled from Fred's Data Set**							
277.	scaffold_300.214923.216626 (matched with frizzled-7 and frizzled homolog 7 and unnamed protein product)	567	432/542(79%)	3/542 (0%)	438/541(80%)	4/541 (0%)	548/553 (99%)	0/553 (0%)
278.	scaffold_1549.10885.12627 (matched with frizzled homolog 8 and frizzled homolog 2 and unnamed protein product)	580	449/665(67%)	110/665(16%)	268/528(50%)	38/528 (7%)	565/581 (97%)	1/581 (0%)
279.	scaffold_536.20133.21884 (matched with frizzled homolog 10 and unnamed protein product)	583	436/577(75%)	3/577 (0%)	449/563(79%)	1/563 (0%)	569/583 (97%)	0/583 (0%)
280.	scaffold_2461.12412.14142 (matched with frizzled homolog 9 and frizzled homolog 10 and unnamed protein product)	576	418/579(72%)	27/579 (4%)	376/560(67%)	11/560 (1%)	474/548 (86%)	16/548 (2%)
281.	scaffold_74.265266.266879 (matched with frizzled homolog 5 and frizzled homolog 2 and unnamed protein product)	537	373/552(67%)	21/552 (3%)	263/512(51%)	37/512 (7%)	504/543 (92%)	6/543 (1%)
282.	scaffold_1909.3949.8009 (matched with frizzled homolog 3 and unnamed protein product)	561	401/610(65%)	76/610 (12%)	408/610(66%)	76/610(12%)	511/582 (87%)	41/582 (7%)
283.	scaffold_43.402294.403766 (matched with frizzled homolog 2 and unnamed protein product)	490	380/483(78%)	21/483 (4%)	387/483(80%)	12/483 (2%)	398/491 (81%)	15/491 (3%)
284.	scaffold_6516.7.1682 (matched with frizzled	553	362/540(67%)	21/540 (3%)	368/536(68%)	18/536 (3%)	422/541	19/541

	homolog 7 and unnamed protein product)						(78%)	(3%)
285.	scaffold_849.34084.38418 (matched with smoothened homolog and unnamed protein product)	714	429/557(77%)	21/557 (3%)	428/559(76%)	22/559 (3%)	480/569 (84%)	27/569 (4%)
286.	scaffold_3606.3197.10767 (matched with frizzled homolog 4 and unnamed protein product)	638	349/507(68%)	32/507 (6%)	349/507(68%)	32/507 (6%)	232/239 (97%)	0/239 (0%)
287.	ABC43433 (odorant receptor, matched with olfactory receptor, family 52, subfamily N, member 5 and similar to olfactory receptor, family 52, subfamily R, member 1 and unnamed protein product)	181	53/145 (36%)	3/145 (2%)	52/160 (32%)	6/160 (3%)	142/156 (91%)	0/156 (0%)
288.	ABC43444 (odorant receptor)	262	No significant similarity found		No significant similarity found		233/254 (91%)	0/254 (0%)
289.	ABC43451 (odorant receptor)	253	No significant similarity found		No significant similarity found		203/240 (84%)	0/240 (0%)
290.	ABF22455 (G protein-coupled receptor 155, matched with G protein-coupled receptor 155 and unnamed protein product)	809	448/800 (56%)	17/800 (2%)	448/801 (55%)	17/801 (2%)	704/819 (85%)	38/819 (4%)
291.	ABF22483 (G protein-coupled receptor 155, matched with G protein-coupled receptor 155)	843	444/827 (53%)	44/827 (5%)	448/819 (54%)	36/819 (4%)	576/733 (78%)	111/733 (15%)
292.	BAA26123 (pheromone receptor, matched with calcium-sensing receptor and unnamed protein product)	868	340/841(40%)	42/841 (4%)	344/844(40%)	49/844 (5%)	493/763 (64%)	27/763 (3%)
293.	BAA26124 (pheromone receptor, matched with calcium-sensing receptor and	864	360/847(42%)	36/847 (4%)	361/846(42%)	35/846 (4%)	691/770 (89%)	0/770 (0%)

	unnamed protein product)							
294.	BAA26125 (pheromone receptor, matched with calcium-sensing receptor and unnamed protein product)	856	352/843(41%)	38/843 (4%)	360/875(41%)	46/875 (5%)	448/478 (93%)	0/478 (0%)
295.	BAA26126 (pheromone receptor matched with calcium-sensing receptor and unnamed protein product)	880	372/857(43%)	32/857 (3%)	367/846(43%)	31/846 (3%)	758/864 (87%)	61/864 (7%)
296.	BAA26127 (pheromone receptor, matched with calcium-sensing receptor and unnamed protein product)	875	324/851(38%)	38/851 (4%)	325/850(38%)	37/850 (4%)	530/647 (81%)	26/647 (4%)
297.	BAA26128 (metabotropic glutamate receptor 1 homologue, matched with glutamate receptor, metabotropic 1 and unnamed protein product)	156	137/156(87%)	0/156 (0%)	138/156(88%)	0/156 (0%)	154/156 (98%)	0/156 (0%)
298.	BAA26129 (metabotropic glutamate receptor 2 homologue, matched with glutamate receptor, metabotropic 2 and glutamate receptor, metabotropic 3 and unnamed protein product)	106	75/106 (70%)	0/106 (0%)	71/106 (66%)	0/106 (0%)	104/106 (98%)	0/106 (0%)
299.	BAA26130 (metabotropic glutamate receptor 7 homologue, matched with glutamate receptor, metabotropic 6 and glutamate receptor, metabotropic 7 and unnamed protein product)	161	122/161(75%)	0/161 (0%)	123/161(76%)	0/161 (0%)	157/172 (91%)	11/172 (6%)
300.	BAA26131 (metabotropic glutamate receptor 8	111	84/111 (75%)	0/111 (0%)	82/111 (73%)	0/111 (0%)	107/111	0/111

	homologue, matched with glutamate receptor, metabotropic 8 and unnamed protein product)						(96%)	(0%)
301.	BAA26134 (pheromone receptor, matched with calcium-sensing receptor and unnamed protein product)	250	132/250(52%)	0/250 (0%)	132/250(52%)	0/250 (0%)	206/211 (97%)	0/211 (0%)
302.	BAA26135 (pheromone receptor, matched with calcium-sensing receptor and unnamed protein product)	250	127/232(54%)	0/232 (0%)	131/234(55%)	4/234 (1%)	235/250 (94%)	0/250 (0%)
303.	BAA26136 (pheromone receptor, matched with calcium-sensing receptor and unnamed protein product)	250	135/249(54%)	0/249 (0%)	136/249(54%)	0/249 (0%)	226/250 (90%)	0/250 (0%)
304.	BAA26137 (pheromone receptor, matched with calcium-sensing receptor and unnamed protein product)	239	116/226(51%)	0/226 (0%)	116/226(51%)	0/226 (0%)	196/226 (86%)	0/226 (0%)
305.	BAA26138 (pheromone receptor, matched with calcium-sensing receptor and unnamed protein product)	250	136/251(54%)	2/251 (0%)	135/251(53%)	2/251 (0%)	243/250 (97%)	0/250 (0%)
306.	BAA26139 (pheromone receptor, matched with calcium-sensing receptor and unnamed protein product)	250	127/234(54%)	0/234 (0%)	128/234(54%)	0/234 (0%)	222/249 (89%)	0/249 (0%)
307.	BAA26140 (pheromone receptors, matched with calcium-sensing receptor and unnamed protein product)	250	124/230(53%)	0/230 (0%)	125/230(54%)	0/230 (0%)	219/250 (87%)	0/250 (0%)
308.	BAA26141 (pheromone receptor, matched with calcium-sensing receptor and unnamed protein product)	251	127/251(50%)	1/251 (0%)	125/251(49%)	1/251 (0%)	230/251 (91%)	0/251 (0%)
309.	BAA26142 (pheromone receptor, matched with calcium-sensing receptor and	251	131/251(52%)	1/251 (0%)	130/251(51%)	1/251 (0%)	231/251 (92%)	0/251 (0%)

	unnamed protein product)							
310.	BAA26143 (pheromone receptor, matched with calcium-sensing receptor and unnamed protein product)	251	130/251(51%)	1/251 (0%)	128/251(50%)	1/251 (0%)	235/251 (93%)	0/251 (0%)
311.	BAA26144 (pheromone receptor, matched with calcium-sensing receptor and unnamed protein product)	251	133/251(52%)	1/251 (0%)	116/227(51%)	1/227 (0%)	231/251 (92%)	0/251 (0%)
312.	BAA26145 (pheromone receptor, matched with calcium-sensing receptor and unnamed protein product)	251	133/251(52%)	1/251 (0%)	132/251(52%)	1/251 (0%)	235/251 (93%)	0/251 (0%)
313.	BAA26146 (pheromone receptor, matched with calcium-sensing receptor and unnamed protein product)	251	120/226(53%)	1/226 (0%)	120/227(52%)	1/227 (0%)	210/220 (95%)	1/220 (0%
314.	BAE78486 (taste receptor, type 1, member 1, matched with taste receptor, type 1, member 1 and unnamed protein product)	845	345/810(42%)	13/810 (1%)	285/685(41%)	13/685 (1%)	771/841 (91%)	0/841 (0%)
315.	CAA09083 (metabotropic glutamate receptor 7, matched with glutamate receptor, metabotropic 7 and unnamed protein product)	551	411/546(75%)	0/546 (0%)	409/546(74%)	0/546 (0%)	542/558 (97%)	11/558 (1%)
316.	NP_001091094 (taste receptor, type 1, member 1, matched with taste receptor, type 1, member1 and unnamed protein product)	845	345/810(42%)	13/810 (1%)	285/685(41%)	13/685 (1%)	771/841 (91%)	0/841 (0%)
